# Ultraconserved element (UCE) probe set design: Base genome and initial design parameters critical for optimization

**DOI:** 10.1002/ece3.5260

**Published:** 2019-06-11

**Authors:** Grey T. Gustafson, Alana Alexander, John S. Sproul, James M. Pflug, David R. Maddison, Andrew E. Z. Short

**Affiliations:** ^1^ Department of Ecology and Evolutionary Biology University of Kansas Lawrence Kansas; ^2^ Biodiversity Institute University of Kansas Lawrence Kansas; ^3^ Department of Anatomy, School of Biomedical Sciences University of Otago Dunedin New Zealand; ^4^ Department of Integrative Biology Oregon State University Corvallis Oregon; ^5^ Department of Biology University of Rochester Rochester New York

**Keywords:** bait design, genomics, phylogenetics, phylogenomics, probe design, ultraconserved elements

## Abstract

Targeted capture and enrichment approaches have proven effective for phylogenetic study. Ultraconserved elements (UCEs) in particular have exhibited great utility for phylogenomic analyses, with the software package phyluce being among the most utilized pipelines for UCE phylogenomics, including probe design. Despite the success of UCEs, it is becoming increasing apparent that diverse lineages require probe sets tailored to focal taxa in order to improve locus recovery. However, factors affecting probe design and methods for optimizing probe sets to focal taxa remain underexplored. Here, we use newly available beetle (Coleoptera) genomic resources to investigate factors affecting UCE probe set design using phyluce. In particular, we explore the effects of stringency during initial design steps, as well as base genome choice on resulting probe sets and locus recovery. We found that both base genome choice and initial bait design stringency parameters greatly alter the number of resultant probes included in final probe sets and strongly affect the number of loci detected and recovered during in silico testing of these probe sets. In addition, we identify attributes of base genomes that correlated with high performance in probe design. Ultimately, we provide a recommended workflow for using phyluce to design an optimized UCE probe set that will work across a targeted lineage, and use our findings to develop a new, open‐source UCE probe set for beetles of the suborder Adephaga.

## 1 INTRODUCTION

The phylogenomics revolution is underway with targeted capture and enrichment approaches (Mamanova et al., 2010; McCormack, Hird, Zellmer, Carstens, & Brumfield, 2013) proving an effective and improved method for phylogenetic studies relative to multilocus Sanger techniques (Blaimer et al., 2015). Among the genomic components that have been targeted, ultraconserved elements (UCEs sensu Faircloth et al., 2012) have demonstrated their utility in reconstructing phylogenies across diverse vertebrate [e.g., fishes (Faircloth, Sorenson, Santini, & Alfaro, 2013), birds (Hosner, Tobias, Braun, & Kimball, 2017; McCormack et al., 2013), reptiles (Crawford et al., 2012; Grismer et al., 2016; Streicher & Wiens, 2017), amphibians (Alexander et al., 2017; Streicher et al., 2018), and mammals (Esselstyn, Oliveros, Swanson, & Faircloth, 2017; McCormack et al., 2012)] and other animal lineages [e.g., cnidarians (Quattrini et al., 2018), arachnids (Starrett et al., 2017), hymenopterans (Faircloth, Branstetter, White, & Brady, 2015), and coleopterans (Baca, Alexander, Gustafson, & Short, 2017; Van Dam et al., 2017)], providing resolution for notoriously difficult‐to‐reconstruct evolutionary histories, such as those of placental mammals (Esselstyn et al., 2017) or the rapid radiation of modern birds (McCormack et al., 2013). Additionally, UCEs are phylogenetically informative at multiple timescales (Faircloth et al., 2012): able to reconstruct both deep (Quattrini et al., 2018) and shallow (Manthey, Campillo, Burns, & Moyle, 2016; Musher & Cracraft, 2018; Smith, Harvey, Faircloth, Glenn, & Brumfield, 2014) evolutionary relationships. Furthermore, UCEs allow for the inclusion of museum specimens (Blaimer, Lloyd, Guillory, & Brady, 2016; McCormack, Tsai, & Faircloth, 2016; Van Dam et al., 2017), even those preserved in formalin (Ruane & Austin, 2017), affording improved taxon sampling by removing limitations associated with specimen preservation style.

Another advantage to the use of UCEs is the ability to develop probe sets that target thousands of orthologous loci across members of a select organismal group, based on low coverage genomic reads from relatively few exemplar taxa (Faircloth, 2017). While universal probe sets designed to work across larger taxonomic groups such as tetrapods (Faircloth et al., 2012; Sun et al., 2014) have proven successful for birds (Hosner et al., 2017; Manthey et al., 2016) and mammals (Esselstyn et al., 2017), it is becoming increasingly apparent that for other diverse animal lineages probe sets tailored to focal taxa result in considerably improved locus recovery. For example, the first Hymenoptera UCE probe set, designed largely using the genome of a single parasitoid wasp species, targeted a total of 1,500 UCE loci, but captured decreasing numbers of loci with increasing phylogenetic distance from this taxon, with one highly divergent study species within Hymenoptera having as few as 341 loci enriched by the probe set (Faircloth et al., 2015). Thus, Branstetter, Longino, Ward, and Faircloth (2017) subsequently developed an expanded Hymenoptera UCE probe set tailored for use in ants, which successfully increased locus capture for target ant taxa, but similarly showed decreased capture rates for more distantly related ants, bees, and wasps. In another example, Faircloth (2017) designed a Coleoptera UCE probe set, primarily using genomes from the beetle suborder Polyphaga. This probe set, targeting 1,172 UCE loci, only recovered 305 loci in 50% of the taxa studied in a phylogenomic analysis of the distantly related beetle suborder Adephaga (Baca et al., 2017). A second investigation by Van Dam et al., 2017 utilized this same probe set for a phylogenomic analysis of weevils (family Curculionidae), members of the same suborder of beetles used during the probe design (Faircloth, 2017). Although capturing considerably more loci (537 loci at 50% data matrix completeness) than the prior study, it still resulted in significantly fewer UCE loci for phylogenetic analysis (368 in the 70% complete data matrix) (Van Dam et al., 2017) than the probe set was designed to provide. This is likely due to the single representative weevil genome used (*Dendroctonus ponderosae*) in the design of the Coleoptera UCE probe set by Faircloth (2017) being only distantly related to those in the Van Dam et al. (2017) study.

In spite of the apparent need for probe sets tailored to focal taxa, factors affecting probe design and methods for optimizing probe sets to focal taxa remain largely underexplored (Branstetter et al., 2017; Glenn & Faircloth, 2016). The need for investigation of factors affecting probe design is made even greater by the increasing number of genomic resources becoming readily available for diverse organisms, and the development of new software enabling probe design.

In this study, we investigate factors affecting probe set design of UCE loci. In particular, we investigate the effect of base genome choice and initial bait design parameters on the resulting probe set, and the number of UCE loci recovered during in silico tests. Currently, there are relatively few thorough workflows or pipelines and associated programs for identifying conserved loci and designing baits targeting them (Campana, 2018; Faircloth, 2017; Johnson et al., 2016; Mayer et al., 2016). We opted to use one of the most widely utilized software packages for UCE phylogenomics, phyluce (Faircloth, 2016), and to follow the workflow outlined in detail by Faircloth (2017) for using phyluce to identify and design baits targeting UCE loci. Based on our results, we provide recommendations and discuss considerations for base genome choice and parameter selections to optimize the design of UCE probe sets using phyluce. Our findings are then applied to develop a new UCE probe set, optimized for beetles of the suborder Adephaga, using newly available genomic resources and incorporating previously published Coleoptera UCE loci.

## 2 MATERIALS AND METHODS

### 2.1 Genomic resources and taxon information

Genomic assemblies used in this study were generated using Illumina reads obtained from seven beetle species from the suborder Adephaga (Table 1) (Pflug, Holmes, Johnston, and Maddison, in prep). Full details are given in Pflug, Holmes, Johnston, and Maddison (in prep); in brief, poor quality reads and adapter sequences were trimmed from Illumina reads using the “Trim Sequences” tool in clc genomics workbench version 9.5.3 (CLC Bio, referred to below as CLC GW), with the trim quality score limit set to 0.5, allowing for a maximum of two ambiguities per read, and searching on both strands to remove adapters, retaining broken pairs. De novo assemblies of paired, trimmed reads in CLC GW were generated using automatic word and bubble size, with the minimum contig length set to 200 bases. Genomic assembly metrics for the genomes used in this study are given in Table 1 (methods for obtaining these metrics are provided in Section 2.9).

**Table 1:** Study taxa and associated genomic assembly information. OSAC, Oregon State Arthropod Collection located at Oregon State University

Species	Voucher # Depository	Family	Estimated read depth	N50	L50	N90	L90	GC%	BUSCO C%
*Bembidion* *haplogonum* Chaudoir	DNA2544 OSAC	Carabidae	58×	591	211,693	231	972,875	30.6	59.3
*Chlaenius sericeus* (Forster)	DNA4821 OSAC	Carabidae	166×	1,765	41,747	408	190,237	29	78.7
*Lionepha* “Waterfalls”	DNA3782 OSAC	Carabidae	8×	5,477	9,102	518	47,974	28.6	68.5
*Pterostichus melanarius* (Illiger)	DNA3787 OSAC	Carabidae	33×	346	216,023	137	864,146	48.8	30.7
*Omoglymmius hamatus* (LeConte)	DNA3783 OSAC	Rhysodidae	13×	370	234,264	143	963,963	50	7.3
*Trachypachus gibbsii* LeConte	DNA3786 OSAC	Trachypachidae	85×	1,758	34,686	213	233,955	32.8	45.8
*Amphizoa insolens* LeConte	DNA3784 OSAC	Amphizoidae	8×	364	160,074	141	668,749	32.3	34.7

Our study species come from four different families within the beetle suborder Adephaga (Table 1). The suborder is diverse, consisting of ~45,000 species (Ślipiński, Leschen, & Lawrence, 2011) exhibiting a wide range of ecologies with both terrestrial and aquatic members. These ecological differences have been used to divide the suborder into two groups: the Hydradephaga containing aquatic members and the Geadephaga for terrestrial species. There has yet to be a comprehensive phylogenetic analysis of the suborder using molecular data, and recent studies with limited taxon sampling have either found these two groups reciprocally monophyletic (McKenna et al., 2015), or with Geadephaga sister to all aquatic families with the exception of the whirligig (the family Gyrinidae) beetles (Baca et al., 2017), or sister to both Gyrinidae and Haliplidae (Zhang et al., 2018). Our study taxa consist largely of families within the Geadephaga with a single hydradephagan representative, *Amphizoa*, belonging to one of the aquatic families consistently supported as sister to the Geadephaga (Baca et al., 2017; McKenna et al., 2015).

The lack of a comprehensive time‐calibrated phylogeny of Adephaga prevents precise estimates of the evolutionary time and phylogenetic distance separating the species in our study. However, the maximum evolutionary time separating our study species is approximately 250 million years, which is similar in age to the estimated split between turtles and archosaurs (Chiari, Cahais, Galtier, & Delsuc, 2012; Shen, Liang, Wen, & Zhang, 2011; Toussaint et al., 2017).

### 2.2 Overview

Using the above genomic resources and the software package phyluce, we developed multiple UCE probe sets while systematically altering key aspects of the design strategy. Throughout this study, we use the term “bait” to refer to the temporary bait sets targeting theoretical conserved loci (an intermediate stage in probe design), with “probe” referring to the final products of probe design (i.e., the set of RNA probes targeting UCE loci that would actually be synthesized, subject to in silico testing). First, we explored the effects of changing which taxon served as the base genome during probe design, a critical decision, as the genomic reads of all other taxa are mapped to this genome in order to identify conserved loci. The second round of experiments focused on the effects of changing the stringency parameters used during temporary bait design in phyluce. Our general workflow for using phyluce to develop probe sets, and the specific methods for our experimentation are provided below. We evaluated the success of the different probe sets by tracking the number of loci identified and/or recovered during the different stages of probe design within phyluce, the number of probes developed, and finally by comparison of the UCE loci recovered during in silico tests. After producing optimized probes, we filtered the probe set to a subset of probes targeting only those loci found within all seven taxa that appear paralogy free. We then used baitstools as an additional check of our final subset of optimized probes in order to identify physical attributes that could affect their synthesis and in vitro utility. Finally, we investigated attributes of base genomes (e.g., assembly metrics and genetic distance from other study taxa) potentially correlated with improved probe design.

### 2.3 Phyluce workflow overview

**Figure 1:**
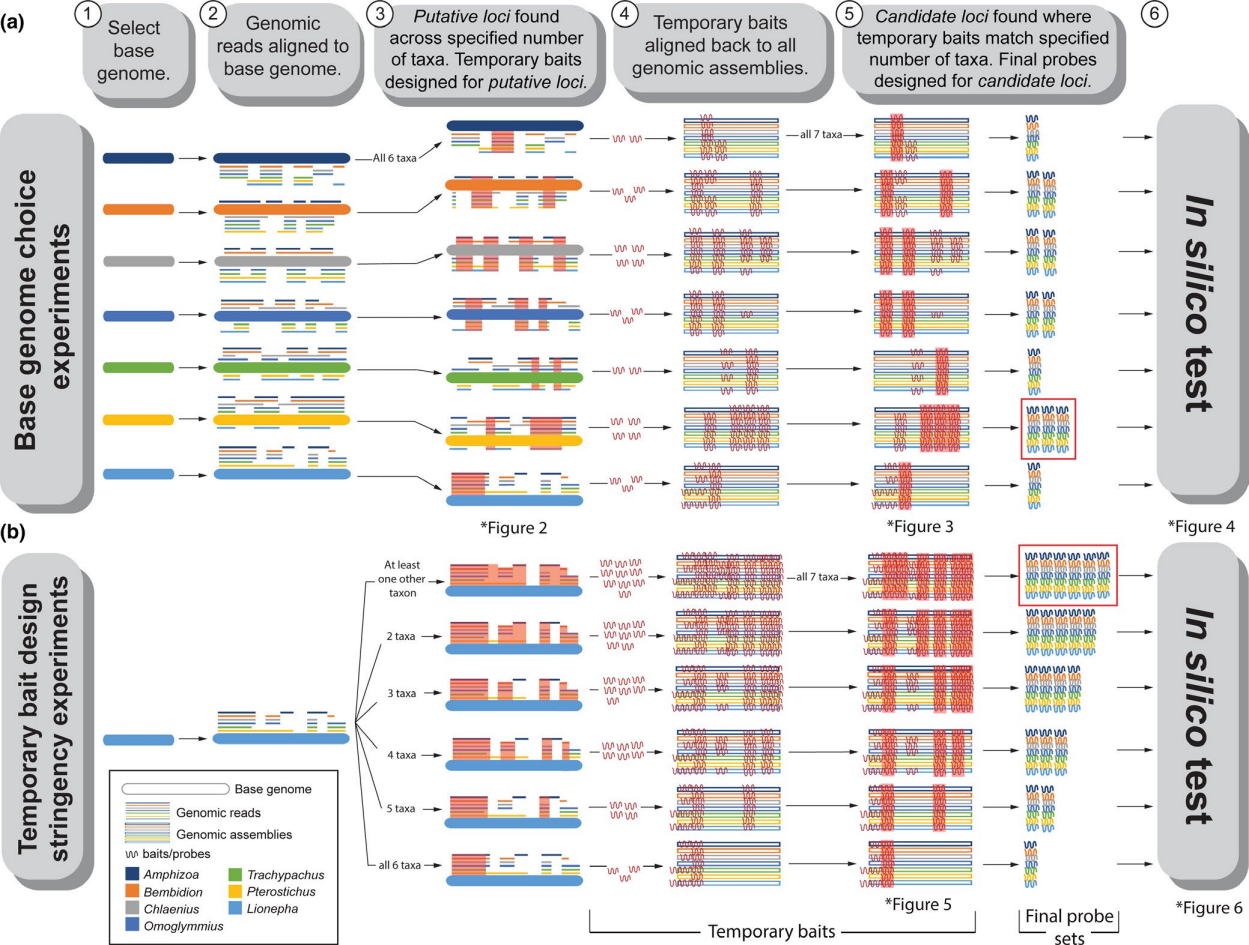
General phyluce workflow for the two sets of probe design experiments conducted using phyluce. Workflow proceeds from left to right with the numbers above text blocks indicating steps referenced in text. (a) depicts the base genome choice experiments described in Section 2.4. (b) depicts the temporary bait design stringency experiments described in Section 2.5. A key for the figure is given in the bottom left corner. “Baits” refer to the temporary bait sets targeting theoretical conserved loci, with “probes” referring to the final products of probe design (i.e., the set of RNA probes targeting UCE loci that would actually be synthesized). Areas highlighted in red show targeted loci/baits that were found across the specified number of taxa. Results depicting the *putative loci* (highly conserved loci shared among taxa), *candidate loci* (loci where temporary baits align across genomes of multiple taxa), and the *recovered loci* (UCE loci recovered during the in silico test) are shown in Figures 2, 3, 4, 5, 6. The red boxes in the final probe set section indicate the combination of parameters leading to the largest number of in silico recovered probes for each set of experiments

A detailed workflow for using phyluce to identify UCE loci and to design probes targeting these loci is described by Faircloth (2017). The general phyluce work flow followed for our experiments is given in Figure 1. The first step in our experiments involved selection of a base genome (Figure 1, step 1) against which genomic reads of the remaining taxa were aligned (Figure 1, step 2). Following removal of duplicate reads, the next step (Figure 1, step 3) identified *putative loci*: highly conserved loci shared among taxa (see Supporting Information for corresponding phyluce terminology and commands). *Putative loci* are those present in the base genome as well as a specified number of additional taxa. The stringency at this step is varied (Figure 1b, step 3) by changing the number of taxa the locus must be found within, ranging from *putative loci* found within the base genome and at least one other taxon (“+1”, the least stringent, designated as “at least one other taxon” on Figure 1b), to those found within the base genome and all remaining taxa (“+6”, the most stringent, designated as “all 6 taxa” on Figure 1b, that is, *putative loci* are defined as those found in the base genome and all six additional taxa). Temporary baits were then developed targeting these *putative loci*. These temporary baits were then aligned back to the genomic assemblies of all study taxa (Figure 1, step 4). This allowed the identification of *candidate loci*: loci for which temporary baits consistently aligned across the genomes of multiple taxa (Figure 1, step 5). By varying the number of taxa within which the candidate loci must be found within, an additional stringency filter can be applied. In the example shown in step 5 of Figure 1, the candidate loci chosen were those for which the temporary baits matched regions in all seven taxa (the base genome plus the other six taxa)—the most stringent option. The final probe set was then created by designing probes customized specifically for each taxon to allow enrichment of the same locus, across the specified number of taxa, using the previously identified *candidate loci*. Finally, an in silico test (Figure 1, step 6) of the resulting probe set was performed using the genomic assemblies of all study taxa in order to assess the number of UCE loci recovered by the final probe set in semirealistic conditions. The purpose of using *recovered loci* (loci recovered in the in silico test) to evaluate the choices involved in probe set design rather than the final probe sets themselves was in part to emulate some of the stochastic variation likely to be seen attempting to recover these loci in vitro.

We applied this workflow to two separate experiments: first, evaluating the use of different taxa as the base genome (Figure 1a, see Section 2.4 for additional methods), and second, varying different parameters for temporary probe design (Figure 1b, see Section 2.5 for additional methods).

### 2.4 Testing the effect of base genome choice on probe design and locus recovery

To test the influence of base genome choice on probe design and locus recovery, we performed the following steps in phyluce (Faircloth, 2016) (as outlined in the phyluce Tutorial IV, available at http://phyluce.readthedocs.io/en/latest/tutorial-four.html). First, a single taxon was selected to serve as the base genome (Faircloth, 2017) (Figure 1a, step 1) and the genomic reads of the remaining taxa were aligned to this genome using stampy (Lunter & Goodson, 2011) (Figure 1a, step 2) under the following mapping parameters: ‐‐maxbasequal 93, ‐‐substitutionrate = 0.05, and insertsize = 400. From the resulting alignment, we identified *putative loci* shared among the base taxon and the remaining study taxa. In this base genome choice experiment, we then proceeded with the most stringent approach to temporary bait design, targeting only those *putative loci* shared across the base genome as well as all six other taxa (Figure 1a, step 3). The influence of modifying the stringency of this step is evaluated by the experiment presented in the next section. We then determined the number of *candidate loci* (where temporary baits consistently aligned across multiple genomes; Figure 1a, step 4). Following this, we took a stringent approach to designing the final probe set by targeting only *candidate loci* found within all seven of the study taxa (the base genome plus all six other taxa) (Figure 1a, step 5). After design of the final probe set, we performed an in silico test (Figure 1a, step 6) of the resulting probes using the genomic assemblies of all the study taxa to determine the number of *recovered loci* by the probe set.

Finally, the UCE loci extracted from each species during the in silico test were aligned using mafft (Katoh & Standley, 2013), ambiguous regions removed via gblocks (Castresana, 2000; Talavera & Castresana, 2007), and a 75% complete matrix generated in phyluce using the command “phyluce_align_get_only_loci_with_min_taxa.” A maximum‐likelihood phylogenetic analysis was performed on this matrix using raxml 8.8.1 (Stamatakis, 2014) and the commands ‐m GTRGAMMA, ‐# autoMRE ‐n BOOT ‐T 24, to investigate any variance among the phylogenetic reconstructions obtained using loci recovered from probe sets designed with different base genomes.

These steps were repeated changing only the taxon that served as the base genome (Figure 1a, step 1).

Following comparison of probe sets designed using different base genomes and their success at locus recovery, we investigated attributes of base genomes potentially correlated with their probe design success. These attributes included genomic assembly metrics such as average estimated read depth, N50, L50, N90, L90, Guanine and Cytosine (GC) percent content, and BUSCO completeness percentage (Simão, Waterhouse, Ioannidis, Kriventseva, & Zdobnov, 2015), as well as average relative genetic distance to the other taxa included in this study. These methods are presented in Sections 2.8 and 2.9.

### 2.5 Testing temporary bait design stringency's influence on probe design and locus recovery

Following investigation into how base genome choice influences probe design and locus recovery, we experimented with relaxing temporary bait design stringency. This involved modifying the step in the phyluce workflow in which temporary baits are constructed based on the identification of *putative loci* shared among the aligned genomic data (Figure 1b, step 3). We incrementally decreased the number of taxa *putative loci* were required to be identified within when designing temporary baits (Figure 1b, step 3). We began first with the most stringent requirement for temporary bait design by targeting *putative loci* found across the base genome and all six other taxa and ended by targeting those found across the base genome and at least one other taxon, the least stringent temporary bait design. We then proceeded to follow the same steps as in Section 2.4 through to the in silico test. It should be emphasized that we maintained the stringent requirement of designing the final probe set based only on *candidate loci* present within all seven study taxa (Figure 1b, step 5). We selected the taxon *Lionepha* to serve as the base genome throughout these tests as it performed moderately well during the base genome choice experiments, being neither the best, nor worst performing genome. After establishing that *Pterostichus* appeared to be the optimum base genome, we also repeated the temporary bait stringency experiment on this base genome, to ensure the results were robust to base genome choice.

### 2.6 Comparison of resulting probe sets

To establish which UCE loci were shared between the probe sets designed using different base genomes in Section 2.4, we used custom bash and R‐scripts to BLAST (at 95% and 99% similarity) between the monolithic files (containing all UCE loci identified across all taxa for each base genome) produced by phyluce (see Alexander 2018a under Data Accessibility for step‐by‐step commands). Loci with single BLAST matches across at least two base genomes for at least one taxon were characterized as: “good” (UCE loci that appeared to be single copy across all taxa across all base genomes), “problematic within taxa” (evidence of potential paralogy within one or more taxa where loci identified within a taxon using one base genome matched to multiple loci within that same taxon identified using a different base genome), and “problematic between taxa” (potential lineage‐specific paralogy: a UCE locus that appeared to be single copy across all base genomes within one taxon appeared to be present in multiple copies in at least one other taxon). We used the “good loci” to identify the base genomes that had the greatest success recovering loci across the seven taxa and that gave the longest alignment in each taxon.

### 2.7 In vitro attributes of final subset of optimized probes

We examined aspects of the final subset of optimized probes designed during this study that could potentially affect synthesis and in vitro utility. We gathered relevant information on the final optimized subset of probes designed during this study relevant to in vitro performance including GC% content, melting temperature (Tm), and sequence complexity, using baitstools (Campana, 2018) and the “checkbaits” command under the following parameters: ‐L 120 ‐c ‐n 25 ‐x 70 ‐q 80 ‐z 120 ‐w.

### 2.8 Genome assembly metrics

We estimated depth of coverage for each genome by mapping reads to the subset of “good” UCE loci (identified using the methods in Section 2.6) that were also found in all seven taxa. The specific reference sequences used for each locus depended on how many base genomes were able to characterize that UCE locus. If a UCE locus was detected only with one base genome, then the UCE locus sequences from this base genome were used as references. If a given locus was recovered using more than one base, then the base genome that gave the longest average alignment across all taxa was selected to provide the reference sequences. We extracted the UCE locus sequences for each taxon using custom bash scripts (see Alexander 2018a under Data Accessibility for step‐by‐step commands). Then, we used GATK v 3.8/4.0, picard, bwa, MAFFT, R, samtools, stringr with custom scripts (see Alexander 2018d under Data Accessibility for step‐by‐step commands) to calculate depth of coverage on a per‐locus basis.

Additional genomic assembly metrics such as N50, L50, N90, L90, Guanine and Cytosine (GC) percent content, and completeness of conserved protein‐coding genes were generated using clc gw v9.5.3 (available from https://www.qiagenbioinformatics.com/), bbmap (Bushnell, 2014) using the stats.sh tool, and BUSCO (Simão et al., 2015).

### 2.9 Estimating relatedness of study taxa

We inferred the relative divergence between the taxa in our study for several markers commonly used in phylogenetic investigations of Coleoptera: 18S, 28S, ArgK, CAD2, CAD4, *wg*, and COI. These markers were obtained either for species included in this study, or for their congeners. Some of these sequences were obtained from the base genomes (CAD2 for *Trachypachus* and *Lionepha*, and COI of all taxa except *Bembidion haplogonum*). The rest were obtained from the literature (Gomez, Will, & Maddison, 2016; Kanda, Pflug, Sproul, Dasenko, & Maddison, 2015; Maddison, 2012; Maddison Baker, & Ober, 1999a, 1999b; Maddison et al., 2009; Maddison & Swanson, 2010; McKenna et al., 2015; Ober, 2002; Sproul & Maddison, 2017), or were newly obtained from Sanger sequencing of PCR products, as specified in Supplemental Information Table S1. Methods for obtaining the new sequences from PCR/Sanger sequencing are given in Maddison (2012). New sequences have been submitted to GenBank with accession numbers MK838494 to MK838511.

We used Geneious^®^ 11.0.5 to calculate average pairwise genetic distances, average proportion of differences (using both Tamura–Nei and HKY corrections), and patristic distances from a neighbor‐joining tree using the Tamura–Nei correction. We ranked taxa from the smallest average genetic distance across markers and methods to the largest average distance.

Additionally, we attempted to extract 50 nuclear protein‐coding markers developed for Coleoptera phylogenetics from genomewide surveys (Che et al., 2017) directly from our study genomes using bait sequences from related taxa available on GenBank via the Zhang et al. (2018) study (Appendix S1). We extracted these nuclear protein‐coding markers from our genomic assemblies using bwa (Li & Durbin, 2009, 2010), data.table (Dowle & Srinivasan, 2017), GATK (McKenna et al., 2010), MAFFT (Katoh & Standley, 2013), picard (available from: http://broadinstitute.github.io/picard/), R (R Core Team, 2017), samtools (Li et al., 2009), and stringr (Wickham, 2017) via custom scripts (see Alexander 2018b under Data Availability for step‐by‐step commands). This was done in an attempt to better approximate divergences of the entire nuclear genomes among our taxa. We then generated relative genetic distance measures among these nuclear protein‐coding markers for our study taxa using EMBOSS distmat (Rice, Longden, & Bleasby, 2000) with a Tamura correction and R via custom scripts (see Alexander 2018c under Data Availability for step‐by‐step commands). Finally, we compared these findings with those of the more commonly used phylogenetic loci represented in our six gene dataset, run through the same distance measures pipeline.

## 3 RESULTS

### 3.1 The effect of base genome choice on probe design and locus recovery

**Figure 2:**
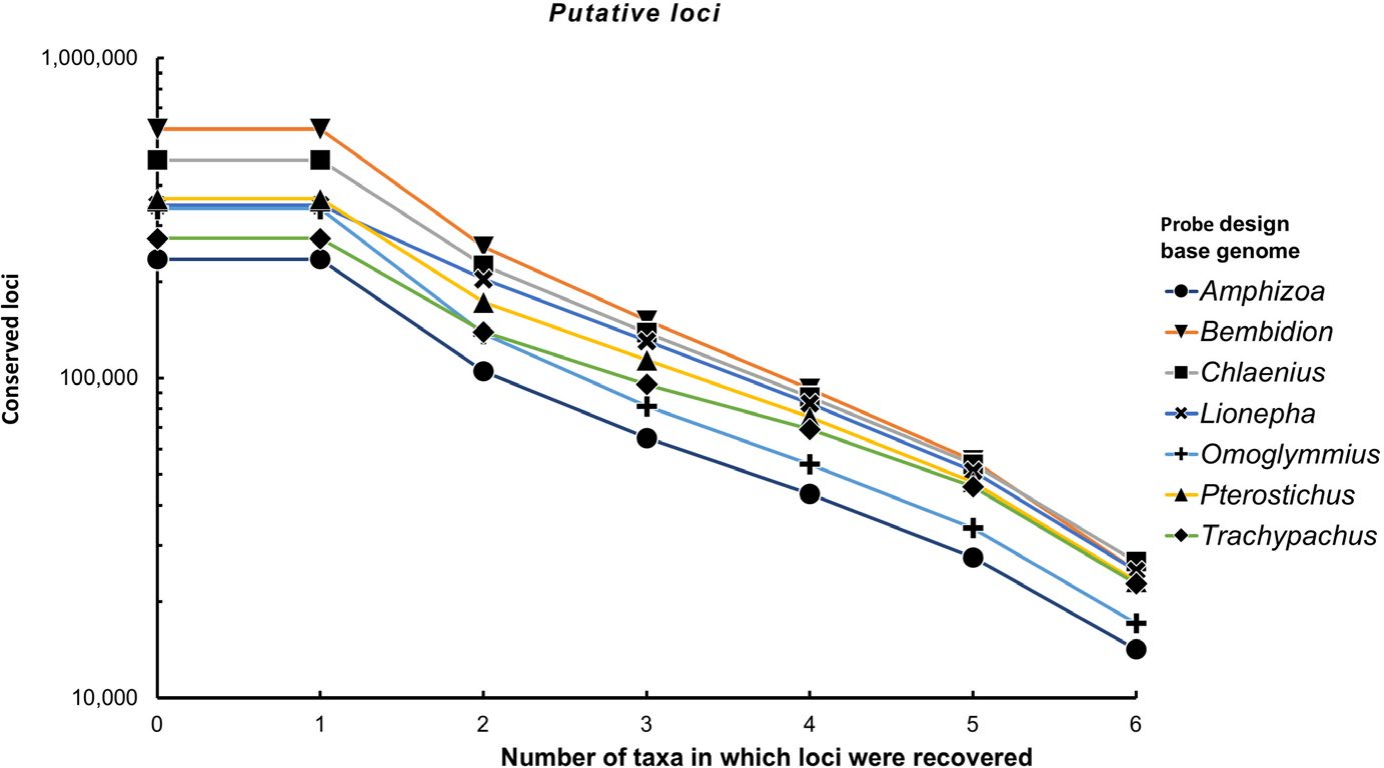
Number of *putative loci* (highly conserved loci shared among taxa) recovered using different base genomes during probe design

**Figure 3:**
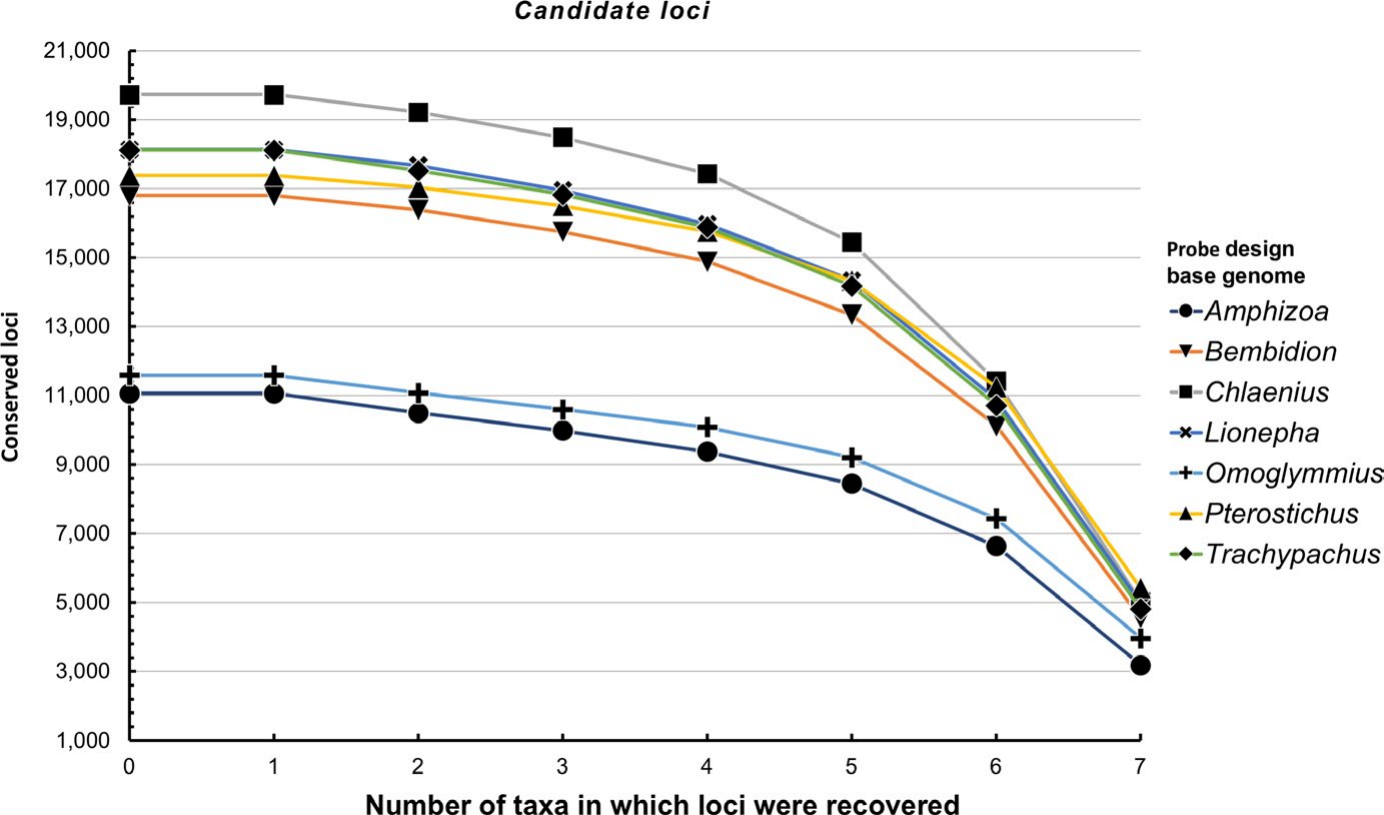
Number of *candidate loci* (loci where temporary baits align across genomes of multiple taxa) shared among different numbers of taxa using different base genomes during probe design

**Figure 4:**
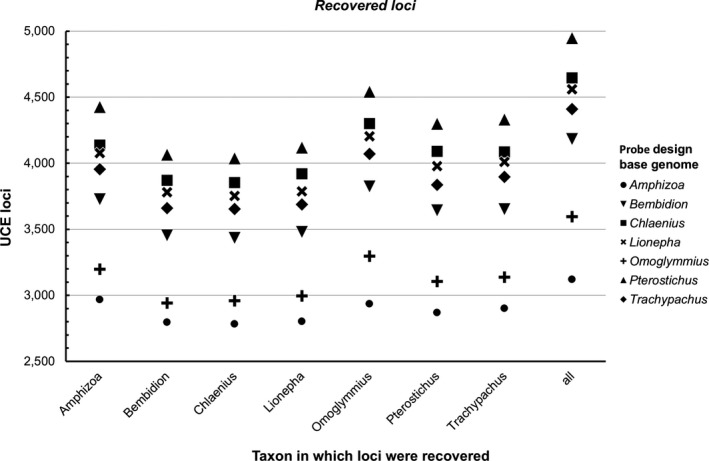
Number of *recovered loci* (UCE loci recovered during the in silico test) shared among different taxa for probe sets designed using different base genomes. All categories provide the sum total of unique loci recovered for that base genome across all taxa

The effect of base genome choice was evident at each stage of the workflow (Figure 1a) from the number of *putative loci* identified (Figure 2, Data S1), to the number of *candidate loci* (Figure 3, Data S2), and finally the number of *recovered loci* during the in silico test (Figure 4, Data S3). There were also consistent trends in the performance of taxa selected to serve as the base genome. For example, the genomes of *Amphizoa* and *Omoglymmius* resulted in the fewest number of *putative loci* (Figure 2), *candidate loci* (Figure 3), and *recovered loci* (Figure 4). These base genomes also resulted in the fewest number of probes designed, as well as the shortest overall sequence lengths recovered across taxa (see Data S2 and S3).

The taxa *Bembidion*, *Lionepha*, and *Trachypachus* performed considerably better as base genomes, resulting in more *putative loci* (Figure 2), *candidate loci* (Figure 3), and *recovered loci* (Figure 4), as well as the design of tens of thousands more probes (Data S2). *Chlaenius* performed even better as the base genome and was the base genome that resulted in the largest number of *putative loci* shared among all six of the other taxa (Figure 2). However, *Chlaenius* did not result in the most *candidate loci* found across all seven taxa (Figure 3) nor *recovered loci* during the in silico test (including *Chlaenius* itself; Figure 4). This suggests the number of *putative loci*, while tracking general trends in a taxon's performance as the base genome, is not an accurate predictor of the best performing base genome for downstream locus detection and recovery.

The best performing base genome was *Pterostichus*. Using *Pterostichus* as the base genome resulted in the largest number of *candidate loci* (Figure 3) found across all seven taxa, and the largest number of *recovered loci* (including the most *recovered loci* for *Pterostichus* itself; Figure 4). The use of *Pterostichus* as a base genome resulted in baits targeting several hundred (i.e., 8%) more loci than the second‐best performing genome, *Chlaenius*, concordantly reflected in the design of several thousand (i.e., ~7%) more probes (Data S2). *Pterostichus* resulted in several hundred (i.e., 6%) more *recovered loci* during the in silico test (Figure 4) than *Chlaenius*, and the resulting concatenated final alignment across taxa was several hundred thousand base pairs (i.e., ~6%) longer than that of *Chlaenius* (Data S3). Despite these differences in performance at recovering and detecting UCE loci, base genome choice did not affect the topology, nor support values of the final maximum‐likelihood phylogenies produced (Figure S1).

### 3.2 Stringency of temporary bait design's effect on probe design and locus recovery

**Figure 5:**
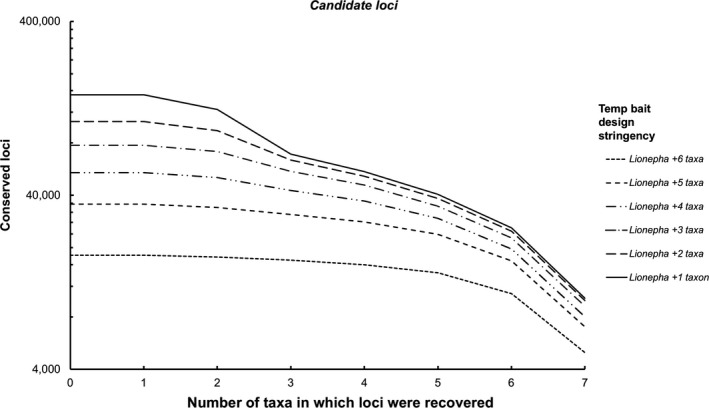
Number of *candidate loci* (loci where temporary baits align across genomes of multiple taxa) found across increasing numbers of taxa for each temporary bait design stringency (requiring loci to be found in the base genome +1 other taxon through to the base genome +6 taxa)

**Figure 6:**
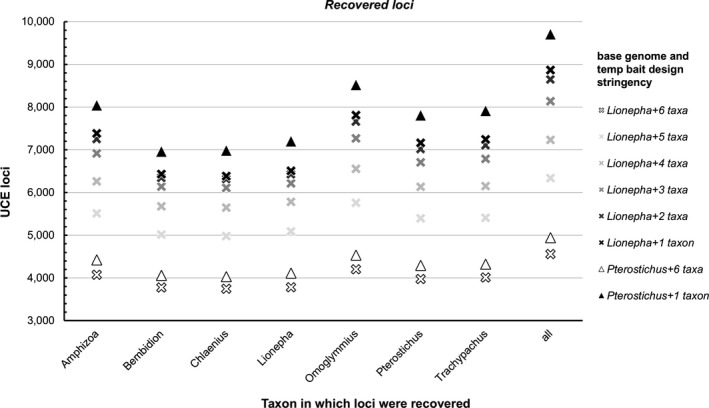
Number of *recovered loci* (UCE loci recovered during the in silico test) in different taxa from probe sets designed using *Lionepha* and *Pterostichus* as base genomes and varying the temporary bait design stringency requirements (requiring loci to be found in the base genome +1 other taxon through to the base genome +6 taxa). The all category provides the sum total of unique loci recovered for that base genome across all taxa

The temporary bait design stringency experiments revealed less stringent parameters for bait design (i.e., requiring sharing of *putative loci* across fewer taxa) greatly improved the number of *candidate loci* identified (Figure 5, Data S4), as well as the number of *recovered loci* during the in silico test (Figure 6, Data S5).

### 3.3 Optimized probe design

The base genome choice experiments identified *Pterostichus* as the optimal base genome. The results from the stringency in temporary bait design experiments showed decreasing the number of taxa *putative loci* were required to be shared across increased both the number of final probes and the number of *recovered loci* during the in silico test (Figure 6). Therefore, to design an initial optimized UCE probe set, we selected *Pterostichus* to serve as the base genome and designed temporary probes under the least stringent requirements, targeting *putative loci* found within the base genome and at least one other taxon.

### 3.4 Comparison of probe sets based on loci found across different probe designs

**Figure 7:**
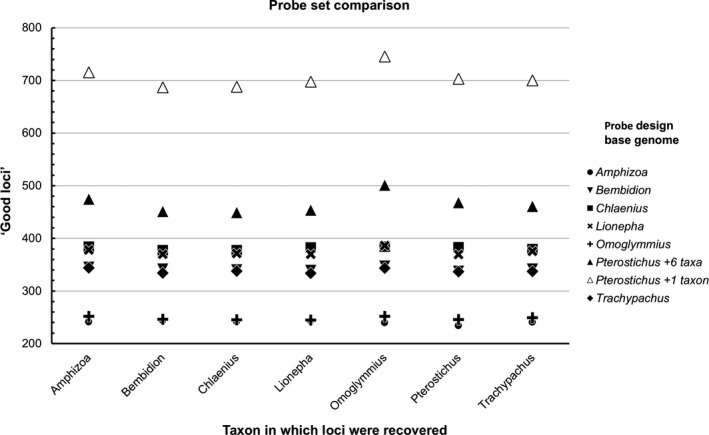
Number of “good loci” (UCE loci that appeared to be single copy across all taxa across all base genomes) recovered in different taxa from probe sets designed using different base genomes

Using the *recovered loci* (i.e., those identified during the in silico test) from the seven base genome choice experiments, as well as the final optimized *Pterostichus *+1 probe design, we identified 8,159 loci through BLAST that matched at 99% similarity across more than one base genome for at least one taxon (95% similarity results revealed very similar patterns, so these results are presented only in Data S6 and S7). Of these, 3,501 “good loci” showed no sign of paralogy within or among taxa across the various base genomes used. Comparisons of base genome performance based on recovery of these “good loci” largely reflected the previously presented results. When *Pterostichus* served as the base genome with temporary baits designed from *putative loci* found in just one other taxon (*Pterostichus *+1 taxon), the largest number of “good loci” were recovered for each taxon in comparison with all other base genomes (Figure 7, Data S8). This pattern persisted even when restricting the comparison to the 2,702 “good loci” detected in all seven taxa and thus does not seem to be an artifact of overrepresentation of loci found only in *Pterostichus* but absent in other lineages (Data S9). We also compared the impact of base genome selection on sequence length of the “good loci” found across all seven study taxa. This could be an important metric, as phylogenetically informative variation in UCEs tends to be greater in the DNA sequences flanking the core conserved element (Faircloth et al., 2012; Van Dam et al., 2017). Longer sequence lengths are therefore more likely to recover these more variable regions. For each “good locus” found in all seven taxa, we recorded the number of taxa where a base genome gave the longest or equally longest sequence lengths across all base genomes. These results again showed that *Pterostichus* (specifically *Pterostichus *+1 taxon) appeared to be the optimal base genome/stringency choice: It resulted in a larger number of loci where it gave the longest alignment for more taxa than the other base genomes (Data S9).

### 3.5 Final optimized probe set

The optimized probe design (*Pterostichus *+1 taxon) resulted in a probe set consisting of 145,599 probes, targeting 11,162 UCE loci. However, synthesizing and enriching this large number of probes and loci would be cost prohibitive for phylogenomic studies with large numbers of taxa. We therefore needed a way to naturally limit the number of probes included in the final probe set. The initial optimized probe set was filtered to a final set of probes targeting the 2,702 “good loci” detected in all seven taxa discussed in Section 3.4. This was done using custom scripts (see Alexander 2018d under Data Accessibility for step‐by‐step commands) and was further limited to only those loci covered by the *Ptersotichus *+1 probes (i.e., the optimized probe design), as 858 loci were derived under other probe design parameters (i.e., different base genomes and temporary bait stringency). Therefore, the final subset of optimized probes targets 2,643 loci that appear paralogy free and are present within all seven study taxa. As part of the final probe set, we also included probes for 305 loci from the previous Coleoptera 1.1Kv1 probe set (Faircloth, 2017) that were successfully recovered during the Baca et al. (2017) study (see Section 4 section below). The final optimized probe set targets a total of 2,948 UCE loci.

In vitro attributes of the final subset of optimized probes (Appendix S2) include a GC% content of all probes between 20% and 70%, with a median of 40.83% and mean of 42%; and a Tm between 80°C and 120°C, with a median of 96.83°C and a mean of 97.69°C. The sequence complexity measure of the probes was very high with a bait linguistic complexity mean and median of 0.99. The GC% content is well within the optimal range for genomic probes (Cruz‐Dávalos et al., 2017; Tewhey et al., 2009) and high‐sequence complexity and Tms increase the likelihood of target specificity.

### 3.6 Assembly metrics of genomic resources used

We used the 2,702 “good” UCE loci found across all seven taxa to estimate coverage depth for the genomes utilized in this study. Genomic read coverage did not appear to correlate with base genome performance (Table 1). *Pterostichus*, the best performing base genome, showed intermediate coverage depth (33×) relative to other taxa (Table 1). The worst performing base genomes, *Omoglymmius* and *Amphizoa*, while estimated as having relatively low coverage depths (13× and 8× respectively), were comparable to that of *Lionepha* (8×) which performed considerably better as a base genome (Figure 4). Finally, *Chlaenius* had the highest estimated coverage depth (166×), yet did not outperform *Pterostichus* which had a much lower coverage depth (33×).

**Figure 8:**
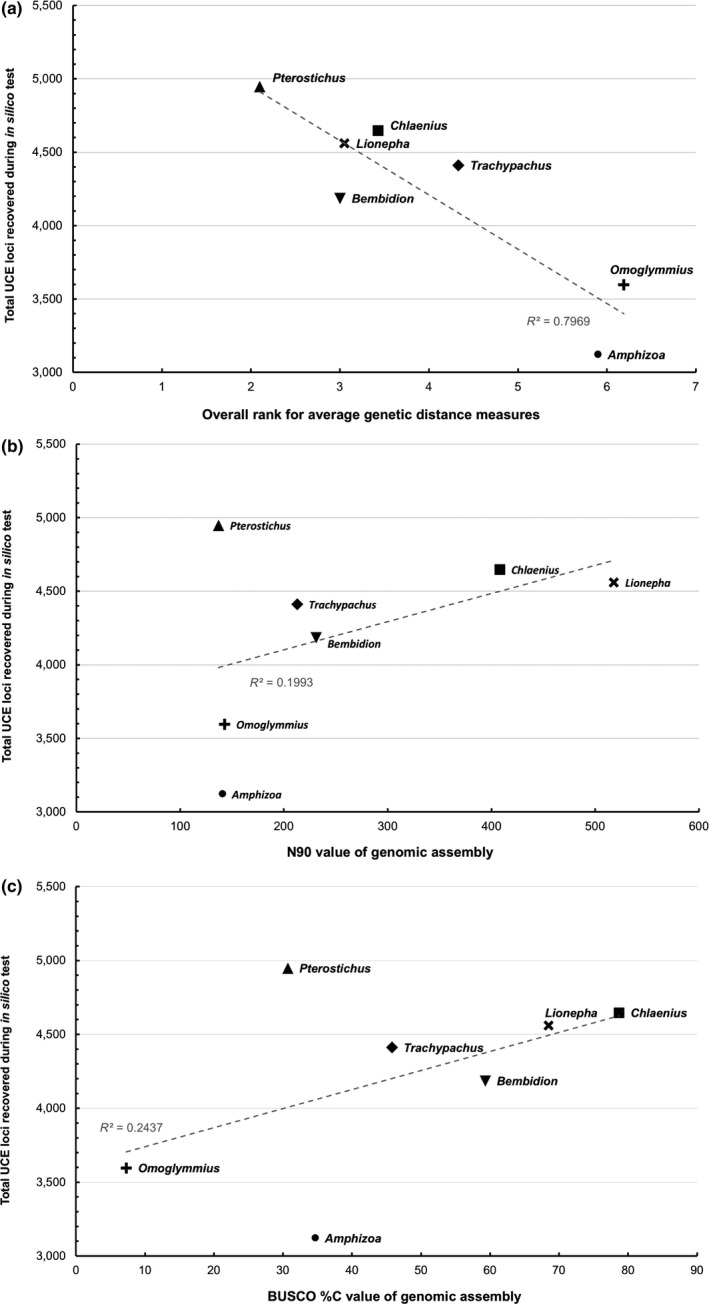
Attributes of study taxa and their genomic assemblies in relation to their performance as base genome. (a) Relationship between average pairwise genetic distance based on six gene fragments regularly use for phylogenetics and total number of UCE loci recovered during in silico test. (b) Relationship between contiguity measure N90 of genomic assembly and total number of UCE loci recovered during in silico test. (c) Relationship between BUSCO %C value of genomic assembly and total number of UCE loci recovered during in silico test

Additional genomic assembly metrics such as N50, L50, N90, L90 identified *Chlaenius*, *Lionepha*, and *Trachypachus* as the best genomic assemblies in terms of contiguity (Figure 8b, Table 1, Data S10), with BUSCO scores (Figure 8c, Table 1, Data S10) supporting the *Chlaenius* genome as being the most complete. Interestingly, *Pterostichus* in addition to *Omoglymmius* and *Amphizoa* had the least complete genomic assemblies and lowest assembly metrics (Figure 8c, Table 1), yet these taxa represent both the best and worst performing base genomes, respectively. Therefore, genomic assembly metrics do not appear to correlate well with base genome performance (Figure 8b,c). Similarly, GC% content of the genome does not appear correlated with suitability as a base genome, in that the best performing base genome, *Pterostichus*, had a comparable GC percentage (nearly 50%, Table 1) to that of one of the worst performing genomes, *Omoglymmius*, while *Amphizoa* (another poorly performing genome) had a GC percentage similar to those of better performing genomes (around 30%, Table 1).

### 3.7 Pairwise genetic distances between taxa

Pairwise genetic distance rankings showed *Pterostichus* as having the smallest genetic distance from all other taxa in the six gene fragments commonly used in phylogenetic studies (28S, COI, CAD2, CAD4, *wg*, ArgK), with *Amphizoa* and *Omoglymmius* the largest genetic distances (Figure 8a, Data S11–S13 for average‐, raw‐, and standardized pairwise genetic distance rankings).

We found average genetic distance ranking based on the six gene dataset to be negatively correlated with a taxon's performance as the base genome as assessed by the total number of UCE loci recovered during in silico testing (Figure 8a), and reflected general trends in genome performance tracked elsewhere (Figures 3, 4, 5).

We attempted to validate the six gene genetic distance measure results with a larger data set by extracting 50 different nuclear protein‐coding loci directly from genomic assemblies of our study taxa (Appendix S1 and Section 2.9). However, this approach proved ineffective due to unevenness in genome assembly quality of our study taxa (Table 1) which resulted in an excess of missing or low‐quality data for taxa with assemblies having a low average depth of coverage (e.g.,* Lionepha*, *Omoglymmius*, and *Amphizoa*). We determined that low coverage and missing data affected data quality for these taxa based on an evident correlation between low sequencing coverage and large genetic distance (Data S14–S19), as well as a maximum‐likelihood phylogenetic analysis of the data set using IQTree (Nguyen, Schmidt, Haeseler, & Minh, 2015), which failed to recover well‐established sister relationships as explained in more detail in Data S20.

## 4 DISCUSSION

Our study using adephagan beetles as an example demonstrates that altering the base genome used during probe design can greatly alter the number of resultant probes included in the final probe set, and more importantly, strongly affects the number of loci detected and recovered during in silico tests. The base genome that recovered the largest number of in silico loci, *Pterostichus*, also performed better at recovering the longest sequences across the seven taxa included in this study. As variable regions flanking core UCEs contain phylogenetically informative sequence data (Faircloth et al., 2012; Van Dam et al., 2017), the ability to recover longer sequences for each locus is likely particularly important for their use in phylogenetic analysis. In addition, we demonstrated that being less stringent during the temporary bait design stage, by focusing on *putative loci* shared across the base genome with as few as one other taxon, resulted in a significantly better final probe set design that detected and recovered more UCE loci during in silico testing.

### 4.1 Considerations for selecting a base genome for probe design

Although we expected at the outset that higher quality base genomes would result in better final probe sets, genomic assembly metrics such as measures of completeness, average read depth, contiguity, and GC content did not appear to correlate well with base genome performance (Figure 8, Table 1). The best performing genome for universal adephagan bait design, *Pterostichus*, had relatively low genomic assembly metrics compared to most other taxa in the study (Table 1). This pattern suggests that genome assembly quality may not be a critical criterion in selecting a base genome. In reporting this finding, we acknowledge that it is possible all our assemblies were above a critical coverage threshold, below which assembly metrics may begin to show stronger correlation with probe design outcomes. Similarly, all our assemblies are limited in quality due to being generated using short‐read Illumina data, and it is possible that assemblies of much higher quality could correlate with greater probe design success. Our results are a useful reference point in that they were generated using a type (e.g., Illumina reads) and quantity (e.g., tens to hundreds of millions of reads) of sequence data that are representative of increasing numbers of unpublished genomes, but the field will benefit from further exploring how assembly quality affects probe design outcomes.

When introducing the workflow for using phyluce to identify and design baits targeting UCE loci, Faircloth (2017) recommends that selection of an appropriate base genome not be focused on ingroup versus outgroup status, but rather annotation and genomic assembly, in order to aid later in selection of probes targeting loci with desirable properties for phylogenetic inference. This is certainly a good recommendation for groups that have well‐annotated genomic assemblies available; however, most invertebrate groups lack such genomic resources, including our study taxa. Furthermore, one of the most powerful aspects of phyluce is its ability to identify and design probe sets for such nonmodel organisms, without the necessity of a complete and annotated genome. Our results demonstrated that UCE probe design can be successfully optimized even while using a base genome whose genomic assembly is of lower coverage and limited quality as phyluce targets conserved regions that are present and shared among the study taxa, rather than searching for a priori known regions to target. We provide a framework for conducting base genome experiments as outlined above (Figure 1) for selecting a base genome in cases where maximizing the number of loci in the probe set is critical. As a less involved alternative to conducting base genome experiments, we provide evidence from a six gene data set (generated through Sanger sequencing) that selecting the taxon with the smallest average genetic distance from the suite of taxa included in probe design to serve as the base genome can correlate with optimal probe design outcomes (Figure 8a). However, our conclusion that genetic distance patterns can guide base genome selection would benefit from further testing given that low sequencing coverage from a subset of our assemblies prevented our validation of this finding with a larger data set (Data S14–S20).

### 4.2 Why does a “relaxed” approach at the temporary bait design stage result in a better probe set?

Perhaps most surprising among the results of our experiments regarding optimal probe set design are those pertaining to temporary bait design stringency. We anticipated a diminishing return in the number of *candidate loci* as the number of taxa *putative loci* were required to share decreased. This is because we expected *putative loci* initially identified as being shared among the base and only one, or a few other taxa, would be unlikely to also be present or detectable in all of the remaining taxa, thus preventing their inclusion as *candidate loci* (the step where we required the baits to target loci found in all seven taxa). However, we did not find this to be the case. Instead, we found that designing temporary baits based on *putative loci* shared among the base genome and as few as one other taxon resulted in significantly better probe design, detecting and recovering more UCE loci, as well as in a better performing probe set. Why this would be the case is not entirely clear. Faircloth, in his informal discussion within the phyluce Tutorial IV (available at: http://phyluce.readthedocs.io/en/latest/tutorial-four.html), suggests loci that are actually present can randomly fail to be detected by phyluce. Perhaps having a temporary bait design protocol with relaxed stringency enables the discovery of such “cryptic” loci shared among taxa. In this case, relaxed temporary bait design would be analogous to casting a wider net which captures both the “cryptic” and initially detected loci, which, when narrowed down later by more stringent parameters applied during final probe design (such as only targeting *candidate loci* present in all study taxa), results in more loci detected and recovered by the final probe set.

It is also possible that this result is an artifact of the two different read mapping programs used by the phyluce pipeline (Faircloth, 2017; Faircloth pers. comm.). Genomic reads are initially aligned to the base genome (Figure 1, step 2) using stampy (Lunter & Goodson, 2011), which has different parameters than that of the lastz (Harris, 2007) alignment used by phyluce when aligning temporary baits back to entire genomic assemblies (Figure 1, step 4). The more permissive LASTZ alignment could therefore potentially be able to detect loci ignored initially by stampy (Faircloth pers. comm.).

### 4.3 In vitro considerations

While we focused here on the optimization of probes based on in silico testing, optimal parameters for in vitro enrichment of UCE probes have not been investigated thoroughly. Certain physical attributes are well known to affect probe efficacy, such as GC% content (Tewhey et al., 2009), annealing temperature (Cruz‐Dávalos et al., 2017), and Gibbs free energy change (delta G) (Matveeva et al., 2016). Additional in‐solution parameters such as buffer stringency (Dabney & Meyer, 2012), washing procedures (Li, Hofreiter, Straube, Corrigan, & Naylor, 2013), and touchdown approaches to hybridization temperature (Li et al., 2013; Paijmans, Fickel, Courtiol, Hofreiter, & Förster, 2016) also affect enrichment of targeted DNA loci. While in silico testing has proven comparable to in vitro testing (Branstetter et al., 2017; Starrett et al., 2017), and the resulting probes designed here should prove suitable for in vitro capture and enrichment (see Section 3.5), there will always be certain loci that remain incalcitrant to enrichment. For this reason, as has previously been emphasized (i.e., Faircloth, 2017), all probe sets should be regarded as experimental until validated through in vitro testing. Future investigation of optimal enrichment parameters of UCE probes will likely help to normalize the number of loci recovered between in silico and in vitro testing.

### 4.4 Tailored UCE probe sets and universal utility

When first introduced, one of the appeals of UCEs was their ability to enrich hundreds to thousands of homologous loci across a clade (Faircloth et al., 2012). To a large extent, UCE probe sets such as those designed for tetrapods (Faircloth et al., 2012) have accomplished this goal with the same probes working well across the major amniote lineages (Crawford et al., 2012; McCormack et al., 2012, 2013; Streicher et al., 2018). However, a probe set with similar universal utility has proven harder to attain for diverse arthropod groups like Coleoptera (see Baca et al., 2017; Van Dam et al., 2017), which while being younger in age (estimated origin during the mid‐Carboniferous (Toussaint et al., 2017)) likely has over ten times the number of species (~380,000 named species; Ślipiński et al., 2011); or Hymenoptera (see Branstetter et al., 2017), a similarly hyperdiverse insect order which may in reality be larger than Coleoptera by as much as 3.2 times (Forbes, Bagley, Beer, Hippee, & Widmayer, 2018).

The first Coleoptera probe set designed by Faircloth (2017) recovered hundreds of loci and provided phylogenetic signal when applied to Adephaga, a suborder of beetles not originally included during probe design (Baca et al., 2017). However, there was considerable conflict between different phylogenetic reconstruction methods with regard to the basal nodes of the phylogeny. Therefore, improved phylogenetic reconstruction of Adephaga would be enhanced by a probe set tailored to this lineage to target more UCE loci and increase phylogenetic resolution. While the probe set developed here was specifically optimized for universal application across Adephaga, our design notably lacked a whirligig beetle genome, as none are currently available. Whirligig beetles (Gyrinidae) comprise ~900 species (Short, 2018), are a relatively old family with an estimated early Triassic origin (Gustafson, Prokin, Bukontaite, Bergsten, & Miller, 2017), and may represent the sister lineage to all other adephagans (Baca et al., 2017). Thus, the probe set designed here could similarly result in fewer loci enriched for members of the Gyrinidae relative to other adephagan families used during probe design. However, this remains to be seen through in vitro testing and, alternatively, this probe set could prove effective across all of Adephaga, preventing need for additional probes tailored to the Gyrinidae.

With an increase in the number of tailored probe sets, concern could be raised that this deflates the value of UCEs as different probe sets result in the recovery of different homologous loci, causing incompatibility between datasets and decreased “universality.” However, UCE probe set development and synthesis affords the ability to mix probes from different designs, diversifying the loci targeted while providing backward compatibility with older data sets (Branstetter et al., 2017). For example, Branstetter et al. (2017) developed a probe set tailored for ant lineages that included newly developed probes targeting ant‐specific loci, while retaining probes from the original universal Hymenoptera probe set designed by Faircloth et al. (2015) that worked well within ant lineages. We similarly follow this approach, and in addition to our newly developed probes (Section 3.5), we include as part of our final probe set 3,667 probes from the prior Coleoptera 1.1Kv1 probe set (Faircloth, 2017) targeting the 305 loci successfully recovered for Adephaga during the Baca et al. (2017) study.

## 5 CONCLUSION

**Figure 9:**
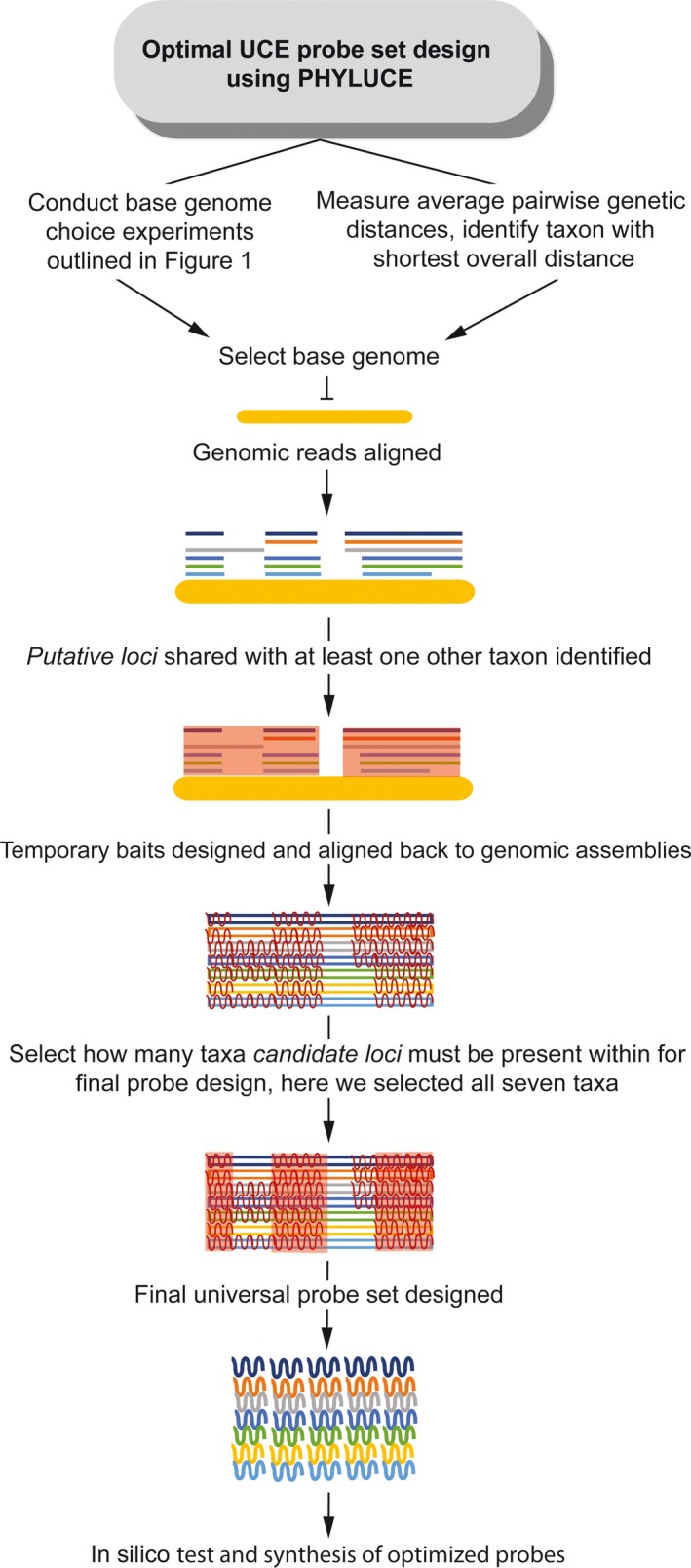
Proposed workflow for designing an optimized universal UCE probe set using phyluce for taxa without a well‐annotated genome available

Given the need to tailor UCE probe sets to focal taxa to improve locus recovery for use in phylogenomic studies and the increasing availability of genomic resources for diverse organisms, information about factors affecting probe design and methods for optimizing locus recovery are becoming increasingly important. We found an optimized UCE probe set depends on selection of an appropriate base genome and beginning probe design with less stringent temporary bait design parameters. We recommend the following workflow (Figure 9) for using phyluce to design an optimized UCE probe set for organisms without a complete and well‐annotated genome available. First, the base genome experiments shown in Figure 1 should be conducted in order to identify an optimal base genome or, alternatively, the base genome can be selected from the taxon with the smallest average genetic distance to the other study taxa as measured by independently generated molecular markers. Then, we recommend that temporary baits are designed based on *putative loci* shared between the base genome and as few as one other taxon. Combined, these factors resulted in an optimized probe set for adephagan beetles, recovering the most UCE loci in in silico tests as well as the longest per‐locus alignments. We make this final optimized adephagan beetle probe set “Adephaga_2.9Kv1,” consisting of 38,948 probes, targeting 2,948 UCE loci, available under a public domain license (CC‐0), similar to the prior Coleoptera probe set designed by Faircloth (2017).

## CONFLICT OF INTEREST

None declared.

## AUTHOR CONTRIBUTIONS

G.T.G., A.A., and A.E.Z.S. conceived of the study. G.T.G. and A.A. conducted probe design and analyses. J.S.S., J.M.P., and D.R.M. obtained and provided the Illumina and Sanger sequence data, assembled the base genomes, and produced genomic assembly metrics. All authors contributed to writing of the manuscript.

## Data Availability

DNA sequences: GenBank MK838494 to MK838511; phyluce BED/BAM files for probe design experiments and Adephaga_2.9Kv1 final probe set fasta file: Dryad https://doi.org/10.5061/dryad.2f62927; Custom scripts: Alexander 2018a: Comparing_monolithic_UCE_fastas available from; https://github.com/laninsky/comparing_monolithic_UCE_fastas; Alexander 2018b: Phase_target available from https://github.com/laninsky/reference_aligning_to_established_loci/tree/master/phase_target; Alexander 2018c: distance_calcs available from: https://github.com/laninsky/distance_calcs; Alexander 2018d: Phase_everyone available from https://github.com/laninsky/reference_aligning_to_established_loci/tree/master/phase_everyone.
