# Supplementary material for: Ultraconserved element (UCE) probe set design: Base genome and initial design parameters critical for optimization
Source: Ecol Evol. 2019 Jun 11;9(12):6933–48. doi: 10.1002/ece3.5260 (PMC6617817; doi:10.1002/ece3.5260)
Supplement: Supplementary file 1 [file ECE3-9-6933-s001.pdf]

Ultraconserved element (UCE) probe set design: base genome and initial design parameters critical for optimization

Grey T. Gustafson[*1,2], Alana Alexander[2,3], John S. Sproul[4,5], James M. Pflug[4], David R. Maddison[4], Andrew E. Z. Short[1,2]

[1]Department of Ecology and Evolutionary Biology, University of Kansas, Lawrence, Kansas, USA

[2]Biodiversity Institute, University of Kansas, Lawrence, Kansas, USA

[3]Department of Anatomy, School of Biomedical Sciences, University of Otago, Dunedin, New Zealand

[4]Department of Integrative Biology, Oregon State University, Corvallis, Oregon, USA

[5]Department of Biology, University of Rochester, Rochester, New York, USA

* corresponding author, Email: gtgustafson@gmail.com

SUPPORTING INFORMATION

Contents

Terminology …………………………………………………………………….... 4

Command line methods ……………………………………………………….…..... 4

Table S1. Genetic Distance Sequences ………………………………………….... 5

Figure S1. Maximum likelihood analyses …………………………………………….. 6

Supporting Data 1. Base genome experiment results: Putative loci ……………………... 7

Supporting Data 2. Base genome experiment results: Candidate loci including locus and probe counts ……………………………………………………………………... 8

Supporting Data 3. Base genome experiment results: Recovered loci including locus counts and alignment length …………………………………………………….. 9

Supporting Data 4. Temporary bait design experiment results: Candidate loci including locus and probe counts ……………………………………………………….. 10

Supporting Data 5. Temporary bait design experiment results: Recovered loci including locus counts and alignment length for both *Lionepha* and *Pterostichus* ………………. 11

Supporting Data 6. Results of the 95% similarity BLAST matching across genomes … 12

Supporting Data 7. Results of the 99% similarity BLAST matching across genomes … 15

Supporting Data 8. Probe set comparison ……………………………………………… 18

Supporting Data 9. Relative locus length of 'good loci' ………………………...…... 19

Supporting Data 10. Full genomic assembly metrics and BUSCO ……...…………….. 20

Supporting Data 11. Average genetic distance rankings based on six gene fragments commonly used in phylogenetic studies …………………………….……………….. 21

Supporting Data 12. Raw genetic distance per locus based on pairwise comparison of all seven species for six gene fragments commonly used in phylogenetic studies ……..…... 23

Supporting Data 13. Standardized genetic distance based on maximum distance per locus between any pair of the seven species for six gene fragments commonly used in phylogenetic studies …………………..………………………………………..……….. 24

Supporting Data 14. Raw genetic distance per locus based on pairwise comparison of nuclear protein-coding loci extracted from genomic assemblies that were found across two or more taxa ……………………………………………………..….………… 25

Supporting Data 15. Raw genetic distance per locus based on pairwise comparison of nuclear protein-coding loci extracted from genomic assemblies that were found across three or more taxa ……….……………………………...………………………….…… 26

Supporting Data 16. Standardized genetic distance based on maximum pairwise distance per locus for nuclear protein coding loci extracted from genomic assemblies that were found across three or more taxa ………………………………...……….………... 27

Supporting Data 17. Raw genetic distance per locus based on pairwise comparison of nuclear protein-coding loci extracted from genomic assemblies that were found across all seven taxa …………………………………………………………….….……… 28

Supporting Data 18. Standardized genetic distance based on maximum pairwise distance per locus for nuclear protein coding loci extracted from genomic assemblies that were found across all seven taxa ……………………………………...……….………... 29

Supporting Data 19. Graphs showing the number of nuclear protein coding genes extracted directly from genomic assemblies, mean raw genetic distance and mean standardized genetic distances estimated from these loci, the locus length, as well as minimum and maximum coverage of the extracted genes. ……….….......................….... 30

Supporting Data 20. Comparison of maximum likelihood phylogenies of loci used for calculating genetic distances 31

Terminology

This paper	Faircloth tutorial
putative loci	*putative conserved loci*
candidate loci	*consistently detected conserved loci*
recovered loci	*recovered loci*

Command line methods

2.4 Testing base genome choice's influence on probe design and locus recovery
Using the command 'phyluce_probe_query_multi_merge_table \' the number of *putative loci* shared among differing numbers of taxa was identified. We used the '--specific-counts 6' subcommand to target loci in the +6 taxa class.

To determine the number of *candidate loci* we used the command 'phyluce_probe_query_multi_fasta_table \'. This command similarly groups the number of targeted loci into classes range from +1 taxon, indicating loci detected only in one taxon, to +7 taxa for loci detected among all seven study taxa (including the base genome). We had PHYLUCE design a final probe set from baits that targeted loci consistently detected in all taxa using the '--specific-counts 7' subcommand.

To observe the number of *recovered loci* specifically for each taxon, and the total number of loci recovered in any given taxon by the probe set during the *in silico* test, the commands 'phyluce_assembly_match_contigs_to_probes \' and 'phyluce_assembly_get_match_counts \' were used respectively.

2.5 Testing temporary bait design stringency's influence on probe design and locus recovery
We incrementally decreased the number of taxa loci were required to be identified within when designing baits by changing the number specified in the subcommand '--specific-counts' of the command 'phyluce_probe_query_multi_merge_table \', beginning with 6 and decreasing to 1. We went from designing temporary baits targeting putative loci identified in the +6 taxa class to those in the +1 taxon class.

2.6 Comparison of resulting probe sets
Monolithic FASTA files were extracted for each base genome using the command 'phyluce_assembly_get_fastas_from_match_counts \' and named according to the taxon used as the base genome during probe design.

Final optimized probe set

For designing a final optimized UCE probe set, we selected *Pterostichus* to serve as the base genome, and followed all of the steps outlined in **Section 2.4** and above, changing the

subcommand ‘--specific-counts’ of the command ‘phyluce_probe_query_multi_merge_table \’ to 1, so temporary baits would target putative loci in the +1 taxon class.

Supplemental Data Table S1. GenBank accession numbers of DNA sequences used to measure genetic distances between taxa. For cells marked with “†”, the DNA sequence was newly obtained from the base genome for that taxon. For cells marked with “*”, the DNA sequence was newly obtained from PCR amplification and Sanger sequencing from a voucher specimen as specified in the GenBank record. All other entries are from previous publications. For those genes not obtained for *Bembidion haplogonum* and *Chlaenius sericeus*, sequences for *Bembidion perspicuum* and *Chlaenius ruficauda*, respectively, were used in their stead.

	**28S**	**18S**	**ArgK**	**CAD2**	**CAD4**	**wg**	**COI**
Amphizoa insolens	EU797339	EU797401	MK838504*	MK838499*	MK838496*	EU797285	MK838508†
Bembidion haplogonum							KU233819
Bembidion perspicuum	GU454740	JN170213	JN170634	KX091941	JN170877	JN171477	
Chlaenius sericeus							MK838511†
Chlaenius ruficauda	AF398680	AF002777	MK838502*	MK838498*	MK838495*	AF398578	
Lionepha 'Waterfalls'	KY246718	KY246684	MK838505*	MK838500†	KY246799	MK838494*	MK838506†
Omoglymmius hamatus	EU797372	AF012520	KP812350	KP812939	KP812939	KP813509	MK838507†
Pterostichus melanarius	AF398707	AF002779	MK118449	EU677533	EU677533	AF398623	MK838510†
Trachypachus gibbsii	EU797394	AF002808	MK838503*	MK838501†	MK838497†	EU797331	MK838509†

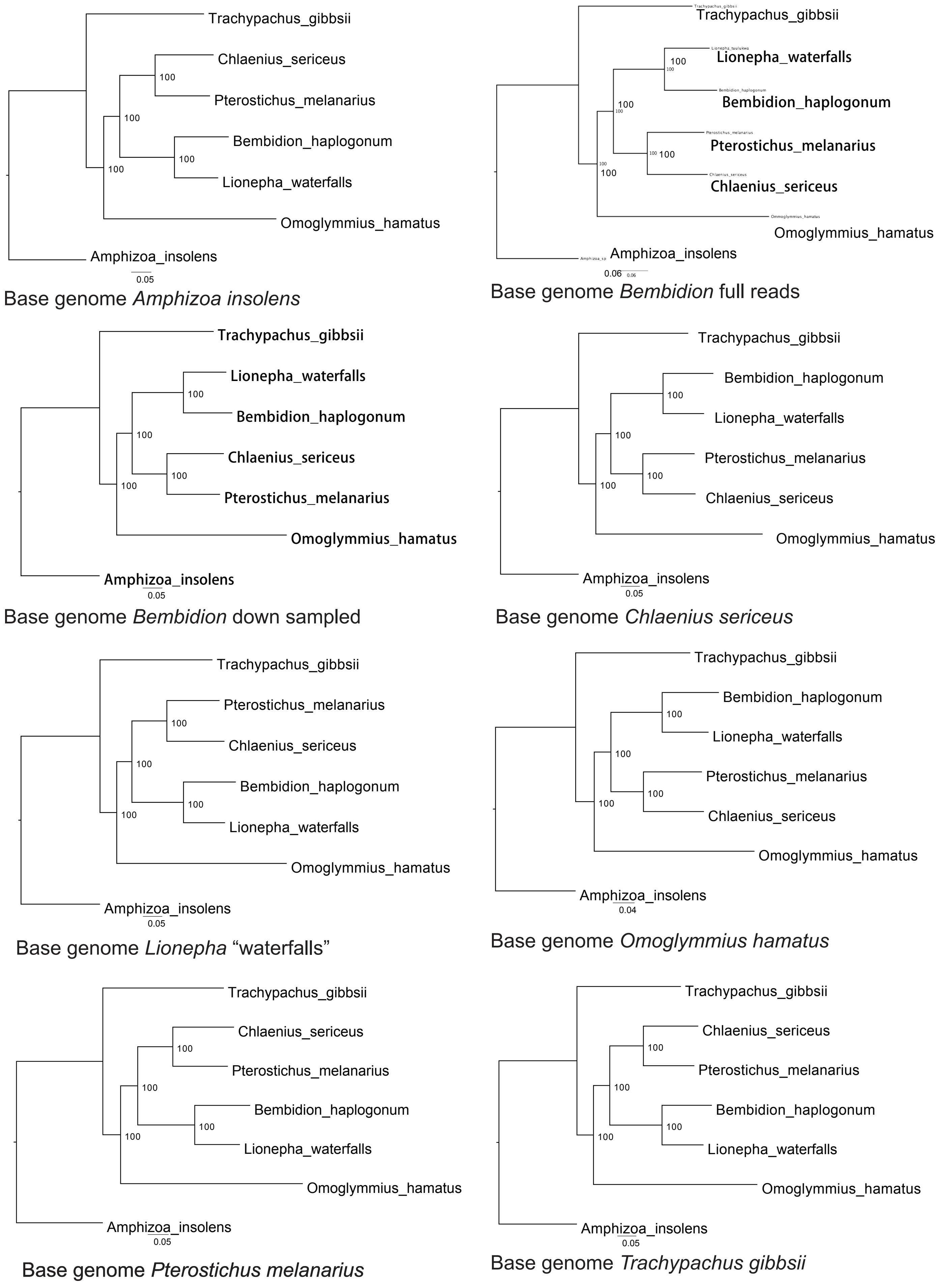

Figure S1. Results of the maximum likelihood analysis using RAxML 8.8.1 on a 75% complete matrix UCE data with ambiguous regions removed. Each tree shows results from probes designed with a different base genome taxon. Number at node indicates bootstrap support.

Supporting Data 1. Base genome experiment results: Putative loci

Number of taxa	Amphizoa	Bembidion	Chlaenius
0	235,013	600,761	480,912
1	235,013	600,761	480,912
2	104,882	257,202	224,792
3	65,052	151,340	138,545
4	43,464	92,509	87,255
5	27,508	55,349	53,876
6	14,163	24,937	26,690

Base genome (columns); Putative loci (values)

Number of taxa	Lionepha	Omoglymmius	Pterostichus	Trachypachus
0	347,428	337,817	362,892	273,030
1	347,428	337,817	362,892	273,030
2	203,516	137,425	172,507	139,397
3	130,477	81,574	113,872	95,513
4	83,299	53,778	75,444	69,131
5	51,250	33,975	47,414	45,808
6	24,985	17,095	23,131	22,772

Base genome (columns); Putative loci (values)

Number of putative loci (highly conserved loci shared among taxa) recovered in different numbers of taxa using different base genomes during probe design. Gray highlights indicate largest number of putative loci recovered for each number of taxa.

Supporting Data 2. Base genome experiment results: Candidate loci including locus and probe counts

Base genome		Amphizoa		Bembidion		Chlaenius
Number of taxa	0	11,067	0	16,807	0	19,735
	1	11,067	1	16,807	1	19,735
	2	10,500	2	16,391	2	19,229
	3	9,991	3	15,758	3	18,491
	4	9,380	4	14,891	4	17,443
	5	8,455	5	13,352	5	15,454
	6	6,641	6	10,127	6	11,423
	7	3,185	7	4,557	7	5,025
locus count		3,185	locus count	4,557	locus count	5,025
probe count		42,430	probe count	61,018	probe count	67,869

Base genome		Lionepha		Omoglymmius		Pterostichus		Trachypachus
Number of taxa	0	18,142	0	11,596	0	17,384	0	18,124
	1	18,142	1	11,596	1	17,384	1	18,124
	2	17,683	2	11,079	2	17,039	2	17,527
	3	16,958	3	10,601	3	16,511	3	16,837
	4	15,976	4	10,089	4	15,763	4	15,882
	5	14,343	5	9,209	5	14,337	5	14,181
	6	10,883	6	7,440	6	11,248	6	10,716
	7	4,981	7	3,966	7	5,421	7	4,816
locus count		4,981	locus count	3,966	locus count	5,421	locus count	4,816
probe count		67,020	probe count	52,990	probe count	72,348	probe count	64,089

Number of candidate loci (loci where temporary baits align across genomes of multiple taxa) shared among different numbers of taxa using different base genomes during probe design. The locus count denotes the total number of candidate loci recovered for all taxa used during probe design with the probe count indicating the total number of temporary baits designed to target these loci. Gray highlights denote the base genome recovering the most loci and resulting in the largest number of temporary baits designed.

Supporting Data 3. Base genome experiment results: Recovered loci including locus and alignment length

Base genome		Amphizoa		Bembidion		Chlaenius
Taxon	Amphizoa	2,969	Amphizoa	3,729	Amphizoa	4,137
	Bembidion	2,796	Bembidion	3,456	Bembidion	3,872
	Claenius	2,784	Claenius	3,437	Claenius	3,855
	Lionepha	2,804	Lionepha	3,482	Lionepha	3,921
	Omoglymmius	2,937	Omoglymmius	3,825	Omoglymmius	4,301
	Pterostichus	2,870	Pterostichus	3,645	Pterostichus	4,090
	Trachypachus	2,902	Trachypachus	3,653	Trachypachus	4,086
	all	3,121	all	4,185	all	4,646
final	loci	2,978	loci	3,736	loci	4,191
alignmen	nucleotides	8,902,723	nucleotides	10,425,539	nucleotides	11,750,069

Base genome		Lionepha		Omoglymmius		Pterostichus		Trachypachus
Taxon	Amphizoa	4,077	Amphizoa	3,198	Amphizoa	4,426	Amphizoa	3,955
	Bembidion	3,781	Bembidion	2,942	Bembidion	4,065	Bembidion	3,661
	Claenius	3,753	Claenius	2,959	Claenius	4,037	Claenius	3,653
	Lionepha	3,788	Lionepha	2,996	Lionepha	4,119	Lionepha	3,688
	Omoglymmius	4,204	Omoglymmius	3,298	Omoglymmius	4,542	Omoglymmius	4,071
	Pterostichus	3,978	Pterostichus	3,105	Pterostichus	4,298	Pterostichus	3,837
	Trachypachus	4,011	Trachypachus	3,139	Trachypachus	4,330	Trachypachus	3,896
	all	4,560	all	3,596	all	4,948	all	4,411
	loci	4,112	loci	3,203	loci	4,452	loci	3,991
	nucleotides	11,415,145	nucleotides	9,216,598	nucleotides	12,414,167	nucleotides	11,235,243

Number of recovered loci (UCE loci recovered during the in silico test) shared among different taxa for probe sets designed using different base genomes. The 'all' category gives the total number of UCE loci recovered. The 'loci' category provides the final number of different loci recovered for all seven taxa. The 'nucleotides' category gives the total alignment length of the recovered data in terms of nucleotide base pairs.

Supporting Data 4. Temporary bait design experiment results: Candidate loci including locus and probe counts

Base genome / Number of taxa	Lionepha +1taxon	Lionepha +2taxa	Lionepha +3taxa	Pterostichus +6taxa
0	151,158	105,958	77,494	17,384
1	151,158	105,958	77,494	17,384
2	124,655	94,222	71,383	17,039
3	69,005	63,722	54,985	16,511
4	54,695	51,408	45,756	15,763
5	40,466	38,388	34,641	14,337
6	25,984	24,868	22,742	11,248
7	10,259	9,924	9,210	5,421
locus count	10,259	9,924	9,210	5,421
probe count	137,396	132,968	123,514	72,348

Base genome / Number of taxa	Lionepha +4taxa	Lionepha +5taxa	Lionepha +6taxa	Pterostichus +1taxon
0	53,923	35,631	18,142	141,169
1	53,923	35,631	18,142	141,169
2	50,652	34,102	17,683	111,959
3	42,721	31,027	16,958	74,479
4	37,083	28,164	15,976	59,857
5	29,495	23,904	14,343	42,881
6	19,646	16,825	10,883	27,500
7	8,017	7,023	4,981	11,162
locus count	8,017	7,023	4,981	11,162
probe count	107,672	94,369	67,020	148,316

Number of candidate loci (loci where temporary baits align across genomes of multiple taxa) found across increasing numbers of taxa for each temporary bait design stringency (requiring loci to be found in the base genome +1 other taxon through to the base genome +6 taxa). The locus count denotes the total number of candidate loci recovered for all taxa used during probe design with the probe count indicating the total number of temporary baits designed to target these loci. Gray highlights denote the base genome and temporary bait design parameters recovering the most loci and resulting in the largest number of temporary baits designed.

Supporting Data 5. Temporary bait design experiment results: Recovered loci including locus counts and alignment length

Base genome		Lionepha +1taxa		Lionepha +2taxa		Lionepha +3taxa		Lionepha +4taxa
Taxon	Amphizoa	7,388	Amphizoa	7,250	Amphizoa	6,918	Amphizoa	6,263
	Bembidion	6,433	Bembidion	6,346	Bembidion	6,138	Bembidion	5,680
	Chlaenius	6,389	Chlaenius	6,314	Chlaenius	6,109	Chlaenius	5,649
	Lionepha	6,509	Lionepha	6,426	Lionepha	6,218	Lionepha	5,785
	Omoglymmius	7,813	Omoglymmius	7,659	Omoglymmius	7,266	Omoglymmius	6,553
	Pterostichus	7,163	Pterostichus	7,020	Pterostichus	6,707	Pterostichus	6,134
	Trachypachus	7,246	Trachypachus	7,107	Trachypachus	6,790	Trachypachus	6,152
	all	8,870	all	8,651	all	8,140	all	7,236
final alignment	loci	7,403	loci	7,272	loci	6,970	loci	6,350
	nucleotides	19,338,078	nucleotides	19,076,372	nucleotides	18,538,922	nucleotides	17,192,439

Base genome		Lionepha +5taxa		Lionepha +6taxa		Pterostichus +1taxa		Pterostichus + 6 taxa
Taxon	Amphizoa	5,514	Amphizoa	4,077	Amphizoa	8,042	Amphizoa	4,426
	Bembidion	5,017	Bembidion	3,781	Bembidion	6,961	Bembidion	4,065
	Chlaenius	4,984	Chlaenius	3,753	Chlaenius	6,984	Chlaenius	4,037
	Lionepha	5,092	Lionepha	3,788	Lionepha	7,196	Lionepha	4,119
	Omoglymmius	5,759	Omoglymmius	4,204	Omoglymmius	8,522	Omoglymmius	4,542
	Pterostichus	5,398	Pterostichus	3,978	Pterostichus	7,807	Pterostichus	4,298
	Trachypachus	5,411	Trachypachus	4,011	Trachypachus	7,914	Trachypachus	4,330
	all	6,340	all	4,560	all	9,706	all	4,948
final alignment	loci	5,569	loci	4,112	loci	8,085	loci	4,452
	nucleotides	15,175,370	nucleotides	11,415,145	nucleotides	20,898,567	nucleotides	12,414,167

Number of recovered loci (UCE loci recovered during the in silico test) in different taxa from probe sets designed using Lionepha and Pterostichus as base genomes and varying the temporary bait design stringency requirements (requiring loci to be found in the base genome +1 other taxon through to the base genome +6 taxa).The 'all' category gives the total number of UCE loci recovered. The 'loci' category provides the final number of different loci recovered for all seven taxa. The 'nucleotides' category gives the total alignment length of the recovered data in terms of nucleotide base pairs. Gray highlights denote the base genome and temporary bait design parameters recovering the most loci and resulting in the largest number of temporary baits designed.

Supporting Data 6. 95% similarity BLAST matching across genomes

Of loci that were found across more than one base genome for at least one taxon

"Good" loci	2967
"Good" loci found in all 7 taxa (across some combiantion of base genom	2275
"Problematic_between" loci	138
"Problematic_between_within" loci	716
"Problematic_within" loci	4316
TOTAL loci	8137

Counts of the number of loci found in betwen 0 and 7 taxa (broken down by base genome used to design probes for that locus)

Of the 2967 good loci: Base genomes --->

Number of taxa locus found in ↓	insilico-Amphizoa-base/ucelocus.txt	insilico-Bembidion-base/ucelocus.txt	insilico-Chlaenius-base/ucelocus.txt	insilico-Lionepha-base/ucelocus.txt	insilico-Omoglymmius-base/ucelocus.txt	insilico-Pterostichus+1-base/ucelocus.txt	insilico-Pterostichus+6-base/ucelocus.txt	insilico-Trachypachus-base/ucelocus.txt
0	2099	1780	1609	1699	2030	722	1366	1744
1	25	65	79	81	55	199	108	84
2	12	38	32	22	39	100	43	23
3	19	35	31	27	28	81	46	27
4	18	22	21	27	15	44	21	25
5	21	23	31	25	24	68	28	27
6	64	74	95	82	61	151	96	101
7	709	930	1069	1004	715	1602	1259	936

By rows: Low number (white) – High number (black)

Interpretation: insilico-Pterostichus+1-base still looks to be the "best" base genome based on this metric. Using it as the base genome led to the largest number of loci found across 1-7 taxa in comparison-

-with other base genomes. It also had the lowest number of times where UCE-probes based on it failed to match to any taxa (despite being found using other base genomes)

Other "goodish" base genomes include Pterostichus+6 and Chlaenius

Conversely, insilico-Amphizoa-base and insilico-Omoglymmius-base seem to be the "worst" base genomes based on this. They had the highest number of failures of probes to find a locus-

-that was found when designed off other base genomes, and the lowest overall numbers for finding these loci in between 1-7 taxa

Counts of the number of loci found in betwen 0 and 7 taxa (broken down by base genome used to design probes for that locus), restricted to loci found in all 7 taxa (across a combination of base genomes)

Of the 2275 good loci found across all 7 taxa: Base genomes --->

Number of taxa locus found in ↓	insilico-Amphizoa-base/ucelocus.txt	insilico-Bembidion-base/ucelocus.txt	insilico-Chlaenius-base/ucelocus.txt	insilico-Lionepha-base/ucelocus.txt	insilico-Omoglymmius-base/ucelocus.txt	insilico-Pterostichus+1-base/ucelocus.txt	insilico-Pterostichus+6-base/ucelocus.txt	insilico-Trachypachus-base/ucelocus.txt
0	1512	1266	1118	1194	1488	518	962	1255
1	4	15	20	18	10	46	14	25
2	3	9	11	3	13	28	5	4
3	6	7	9	4	6	15	4	6
4	4	5	7	10	5	10	3	6
5	11	7	8	7	14	19	4	12
6	26	36	33	35	24	37	24	31
7	709	930	1069	1004	715	1602	1259	936

By rows: Low number (white) – High number (black)

Supporting Data 6. 95% similarity BLAST matching across genomes

Interpretation When constraining to just loci found across all 7 taxa, the Pterostichus bases look to be best. They are found in all 7 taxa to the greatest degree among the other base genomes, and probes based-
-on them fail to be found in taxa the least amount out of all the base-genomes used
Amphizoa, Bembidion and Omoglymmius again seem to be the worst - they have the largest number of loci where probes based on them fail to recover the loci in any taxa, and the lowest-
-numbers of loci where probes designed on them are found in all 7 taxa

Counts of the number of loci where that base genome led to the longest alignment (in up to 7 taxa)

Of the 2967 good loci: Base genomes --->

Number of taxa base genome gave longest alignment in ↓	insilico-Amphizoa-base/ucelocus.txt	insilico-Bembidion-base/ucelocus.txt	insilico-Chlaenius-base/ucelocus.txt	insilico-Lionepha-base/ucelocus.txt	insilico-Omoglymmius-base/ucelocus.txt	insilico-Pterostichus+1-base/ucelocus.txt	insilico-Pterostichus+6-base/ucelocus.txt	insilico-Trachypachus-base/ucelocus.txt
0	2164	1870	1830	2015	2332	1296	2405	2186
1	166	231	272	262	189	439	145	264
2	181	220	173	182	137	259	62	158
3	145	221	216	158	109	216	45	133
4	139	158	159	124	79	203	28	101
5	81	121	132	95	50	149	35	75
6	55	87	109	80	42	146	49	37
7	36	59	76	51	29	259	198	13

By rows
Low number
High number

Interpretation: Probes based on Pterostichus+1 seem to do the best job recovering loci of the longest length during the in silico test, followed by
Pterostichus+6 and Chlaenius. These base genomes also have the lowest number of loci where they failed to give the longest alignment
in any taxa
Amphizoa and Omoglymmius seem to do the worst at recovering the longest loci (and have the largest number of loci where they failed to
give the longest alignment in any taxa). Bembidion, Lionepha and Trachpachus are kind of middling

Counts of the number of loci where that base genome led to the longest alignment (in up to 7 taxa), restricted to loci found in all 7 taxa (across a combination of base genomes)

Of the 2275 good loci found across all 7 taxa: Base genomes --->

Number of taxa base genome gave longest alignment in ↓	insilico-Amphizoa-base/ucelocus.txt	insilico-Bembidion-base/ucelocus.txt	insilico-Chlaenius-base/ucelocus.txt	insilico-Lionepha-base/ucelocus.txt	insilico-Omoglymmius-base/ucelocus.txt	insilico-Pterostichus+1-base/ucelocus.txt	insilico-Pterostichus+6-base/ucelocus.txt	insilico-Trachypachus-base/ucelocus.txt
0	1567	1332	1311	1472	1750	1011	1886	1627
1	137	165	199	192	143	270	73	215
2	158	181	144	149	107	177	33	133
3	125	200	181	140	96	160	24	108
4	126	145	145	111	70	170	19	88
5	74	113	119	85	43	118	20	61
6	52	80	100	75	37	110	22	30
7	36	59	76	51	29	259	198	13

By rows
Low number
High number

Interpretation: Same interpretation as above

Supporting Data 6. 95% similarity BLAST matching across genomes

Data broken down by taxa. If a base genome recovered the locus for a given taxa, it was divided by the total number of base genomes that managed to recover the locus for that taxa (to standardize by the total number of loci found for that taxa)

Base genomes --->

Taxa loci recovered in

Of the 2967 good loci:	insilico-Amphizoa-base/ucelocus.txt	insilico-Bembidion-base/ucelocus.txt	insilico-Chlaenius-base/ucelocus.txt	insilico-Lionepha-base/ucelocus.txt	insilico-Omoglymmius-base/ucelocus.txt	insilico-Pterostichus+1-base/ucelocus.txt	insilico-Pterostichus+6-base/ucelocus.txt	insilico-Trachypachus-base/ucelocus.txt	Total number of loci out of 12994 "good" loci
**Amphizoa**	203.005	286.205	330.938	312.883	211.871	602.3	407.374	294.424	2445.995
**Bembidion**	204.579	279.921	323.036	301.679	203.662	579.7286	387.41	285.986	2361.421
**Chlaenius**	202.432	280.192	321.401	303.561	201.327	578.3845	385.782	288.92	2359.568
**Lionepha**	204.446	280.056	327.132	304.132	201.415	588.7321	389.28	286.806	2377.554
**Omoglymmius**	203.226	290.498	330.09	320.224	209.819	641.9429	435.39	294.81	2522.774
**Pterostichus**	196.3	279.636	328.352	302.593	205.221	594.4857	402.11	291.302	2403.7
**Trachypachus**	203.896	282.306	326.28	309.713	205.501	590.0393	394.473	289.792	2398.104
**TOTAL**	1417.88	1978.81	2287.23	2154.78	1438.82	4175.613	2801.82	2032.04	

Interpretation: insilico-Pterostichus+1 again seems to be the best base genome across taxa, with Amphizoa/omoglymmius the worst

Data broken down by taxa. If a base genome recovered the locus for a given taxa, it was divided by the total number of base genomes that managed to recover the locus for that taxa (to standardize by the total number of loci found for that taxa)

Base genomes --->

Taxa loci recovered in

Of the 2275 good loci found across all 7 taxa:	insilico-Amphizoa-base/ucelocus.txt	insilico-Bembidion-base/ucelocus.txt	insilico-Chlaenius-base/ucelocus.txt	insilico-Lionepha-base/ucelocus.txt	insilico-Omoglymmius-base/ucelocus.txt	insilico-Pterostichus+1-base/ucelocus.txt	insilico-Pterostichus+6-base/ucelocus.txt	insilico-Trachypachus-base/ucelocus.txt	Total number of loci out of 12994 "good" loci
**Amphizoa**	179.252	251.943	287.512	275.505	181.11	500.4548	348.562	250.662	2095.748
**Bembidion**	180.736	252.329	288.152	273.036	182.236	500.4024	349.417	248.693	2094.264
**Chlaenius**	180.015	251.225	287.435	275.627	180.894	500.4345	348.665	250.704	2094.985
**Lionepha**	180.137	250.08	288.906	272.439	181.032	502.806	350.187	249.413	2094.863
**Omoglymmius**	178.157	251.1	288.495	274.326	179.371	506.2286	350.543	246.779	2096.843
**Pterostichus**	178.417	250.736	288.519	271.51	179.171	505.3524	351.543	249.752	2096.583
**Trachypachus**	179.044	251.177	286.904	275.768	180.923	503.6774	349.444	248.063	2095.956
**TOTAL**	1255.76	1758.59	2015.92	1918.21	1264.74	3519.356	2448.36	1744.07	

Interpretation: insilico-Pterostichus+1 again seems to be the best base genome across taxa, with omoglymmius the worst

Base genomes --->

By rows, except for vertical total

Low number

High number

Supporting Data 7. 99% similarity BLAST matching across genomes

Of loci that were found across more than one base genome for at least one taxon

"Good" loci	3501
"Good" loci found in all 7 taxa (across some combiantion of base genomes)	2702
"Problematic_between" loci	194
"Problematic_between_within" loci	686
"Problematic_within" loci	3778
TOTAL loci	8159

By rows

Low number

High number

Counts of the number of loci found in betwen 0 and 7 taxa (broken down by base genome used to design probes for that locus)

Of the 3501 good loci: Base genomes --->

Number of taxa locus found in ↓	insilico-Amphizoa-base/ucelocus.txt	insilico-Bembidion-base/ucelocus.txt	insilico-Chlaenius-base/ucelocus.txt	insilico-Lionepha-base/ucelocus.txt	insilico-Omoglymmius-base/ucelocus.txt	insilico-Pterostichus+1-base/ucelocus.txt	insilico-Pterostichus+6-base/ucelocus.txt	insilico-Trachypachus-base/ucelocus.txt
0	2476	2080	1919	1976	2378	858	1654	2075
1	28	81	89	89	65	232	120	98
2	15	48	38	28	48	112	44	28
3	25	41	35	37	33	100	51	38
4	19	23	27	32	18	55	24	26
5	27	29	41	42	30	89	37	35
6	71	87	106	93	78	172	110	114
7	840	1112	1246	1204	851	1883	1461	1087

Interpretation: insilico-Pterostichus+1-base still looks to be the "best" base genome based on this metric. Using it as the base genome led to the largest number of loci found across 1-7 taxa in comparison with other base genomes. It also had the lowest number of times where UCE-probes based on it failed to match to any taxa (despite being found using other base genomes)

Other "goodish" base genomes include Pterostichus+6 and Chlaenius

Conversely, insilico-Amphizoa-base and insilico-Omoglymmius-base seem to be the "worst" base genomes based on this. They had the highest number of failures of probes to find a locus that was found when designed off other base genomes, and the lowest overall numbers for finding these loci in between 1-7 taxa

Counts of the number of loci found in betwen 0 and 7 taxa (broken down by base genome used to design probes for that locus), restricted to loci found in all 7 taxa (across a combination of base genomes)

Of the 2702 good loci found across all 7 taxa: Base genomes --->

Number of taxa locus found in ↓	insilico-Amphizoa-base/ucelocus.txt	insilico-Bembidion-base/ucelocus.txt	insilico-Chlaenius-base/ucelocus.txt	insilico-Lionepha-base/ucelocus.txt	insilico-Omoglymmius-base/ucelocus.txt	insilico-Pterostichus+1-base/ucelocus.txt	insilico-Pterostichus+6-base/ucelocus.txt	insilico-Trachypachus-base/ucelocus.txt
0	1797	1494	1345	1399	1756	620	1172	1506
1	5	19	26	24	12	60	21	33
2	4	13	13	4	17	32	5	7
3	8	10	12	7	8	20	5	7
4	5	6	8	11	8	14	4	6
5	13	7	14	14	15	28	7	15
6	30	41	38	39	35	45	27	41
7	840	1112	1246	1204	851	1883	1461	1087

Supporting Data 7. 99% similarity BLAST matching across genomes

Interpretation When constraining to just loci found across all 7 taxa, the Pterostichus bases look to be best. They are found in all 7 taxa to the greatest degree among the other base genomes, and probes based on them fail to be found in taxa the least amount out of all the base-genomes used

Amphizoa and Omoglymmius seem to be the worst - they have the largest number of loci where probes based on them fail to recover the loci in any taxa, and the lowest numbers of loci where probes designed on them are found in all 7 taxa

Counts of the number of loci where that base genome led to the longest alignment (in up to 7 taxa)

Of the 3501 good loci: Base genomes --->

Number of taxa base genome gave longest alignment in ↓	insilico-Amphizoa-base/ucelocus.txt	insilico-Bembidion-base/ucelocus.txt	insilico-Chlaenius-base/ucelocus.txt	insilico-Lionepha-base/ucelocus.txt	insilico-Omoglymmius-base/ucelocus.txt	insilico-Pterostichus+1-base/ucelocus.txt	insilico-Pterostichus+6-base/ucelocus.txt	insilico-Trachypachus-base/ucelocus.txt
0	2553	2197	2168	2359	2724	1535	2862	2587
1	194	260	314	304	236	513	155	315
2	204	256	214	212	159	312	70	196
3	182	262	245	194	138	260	50	148
4	157	196	185	158	97	228	30	116
5	104	145	162	122	66	181	45	82
6	65	116	125	95	48	170	57	39
7	42	69	88	57	33	302	232	18

Interpretation: Probes based on Pterostichus+1 seem to do the best job recovering loci of the longest length during the in silico test, followed by Pterostichus+6 and Chlaenius. These base genomes also have the lowest number of loci where they failed to give the longest alignment in any taxa

Amphizoa and Omoglymmius seem to do the worst at recovering the longest loci (and have the largest number of loci where they failed to give the longest alignment in any taxa). Bembidion, Lionepha and Trachpachus are kind of middling

Counts of the number of loci where that base genome led to the longest alignment (in up to 7 taxa), restricted to loci found in all 7 taxa (across a combination of base genomes)

Of the 2702 good loci found across all 7 taxa: Base genomes --->

Number of taxa base genome gave longest alignment in ↓	insilico-Amphizoa-base/ucelocus.txt	insilico-Bembidion-base/ucelocus.txt	insilico-Chlaenius-base/ucelocus.txt	insilico-Lionepha-base/ucelocus.txt	insilico-Omoglymmius-base/ucelocus.txt	insilico-Pterostichus+1-base/ucelocus.txt	insilico-Pterostichus+6-base/ucelocus.txt	insilico-Trachypachus-base/ucelocus.txt
0	1860	1577	1564	1739	2055	1200	2250	1944
1	161	185	233	226	183	326	80	258
2	177	211	181	171	124	217	39	160
3	161	237	206	171	120	193	27	119
4	142	179	169	140	86	192	21	102
5	97	136	146	109	58	143	27	69
6	62	108	115	89	43	129	26	32
7	42	69	88	57	33	302	232	18

Interpretation: Same interpretation as above

Supporting Data 7. 99% similarity BLAST matching across genomes

Data broken down by taxa. If a base genome recovered the locus for a given taxa, it was divided by the total number of base genomes that managed to recover the locus for that taxa (to standardize by the total number of loci found

Base genomes ---> Of the 3501 good loci:	insilico-Amphizoa-base/ucelocus.txt	insilico-Bembidion-base/ucelocus.txt	insilico-Chlaenius-base/ucelocus.txt	insilico-Lionepha-base/ucelocus.txt	insilico-Omoglymmius-base/ucelocus.txt	insilico-Pterostichus+1-base/ucelocus.txt	insilico-Pterostichus+6-base/ucelocus.txt	insilico-Trachypachus-base/ucelocus.txt	TOTAL
**amphizoa**	241.357	345.755	384.188	378.19	252.126	716.11	474.35	343.924	2894.643
**bemHap1**	243.832	342.256	377.72	369.944	246.201	687.506	450.987	334.554	2809.168
**chlSer1**	241.58	340.704	377.77	371.801	245.492	688.296	449.185	338.173	2811.42
**lioTuu1**	243.549	339.515	382.732	369.913	244.87	698.058	453.473	333.889	2822.451
**omoHam1**	239.549	347.785	384.351	386.532	252.094	746.156	500.937	343.596	2961.451
**pterMel1**	234.219	338.029	383.419	369.457	245.693	703.529	467.736	336.919	2844.781
**traGib1**	240.535	341.975	379.899	375.546	249.532	700.501	460.651	337.361	2845.465
**TOTAL**	1684.62	2396.02	2670.08	2621.38	1736.01	4940.16	3257.32	2368.42	

(Row label axis: Taxa loci recovered in)

Interpretation: insilico-Pterostichus+1 again seems to be the best base genome across taxa, with Amphizoa/omoglymmius the worst

Data broken down by taxa. If a base genome recovered the locus for a given taxa, it was divided by the total number of base genomes that managed to recover the locus for that taxa (to standardize by the total number of loci found for that taxa)

Base genomes ---> Of the 2702 good loci found across all 7 taxa:	insilico-Amphizoa-base/ucelocus.txt	insilico-Bembidion-base/ucelocus.txt	insilico-Chlaenius-base/ucelocus.txt	insilico-Lionepha-base/ucelocus.txt	insilico-Omoglymmius-base/ucelocus.txt	insilico-Pterostichus+1-base/ucelocus.txt	insilico-Pterostichus+6-base/ucelocus.txt	insilico-Trachypachus-base/ucelocus.txt	TOTAL
**amphizoa**	213.938	304.626	336.512	331.979	217.248	595.731	407.671	294.295	2488.062
**bemHap1**	215.623	305.846	337.087	331.351	220.042	592.646	406.961	292.444	2486.377
**chlSer1**	215.08	304.12	336.304	334.285	218.108	594.396	406.285	293.423	2486.92
**lioTuu1**	214.823	303.206	338.923	330.137	218.654	595.965	407.546	292.746	2487.177
**omoHam1**	212.563	303.437	338.173	332.635	216.613	599.825	409.306	289.449	2489.437
**pterMel1**	213.136	303.895	337.919	329.34	216.41	600.029	409.802	291.469	2488.864
**traGib1**	213.849	303.846	336.023	332.768	219.12	598.139	407.623	290.632	2488.151
**TOTAL**	1499.01	2128.98	2360.94	2322.49	1526.19	4176.73	2855.19	2044.46	

(Row label axis: Taxa loci recovered in)

Interpretation: insilico-Pterostichus+1 again seems to be the best base genome across taxa, with omoglymmius the worst

By rows

(white)	Low number
(black)	High number

Supporting Data 8. Probe set comparison

Base genome: Amphizoa, Bembidion, Chlaenius, Lionepha (Taxon loci recovered in)

Base genome	Amphizoa		Bembidion		Chlaenius		Lionepha
Amphizoa	241.357	Amphizoa	345.754	Amphizoa	384.188	Amphizoa	378.19
Bembidion	243.832	Bembidion	342.255	Bembidion	377.72	Bembidion	369.944
Chlaenius	241.579	Chlaenius	340.703	Chlaenius	377.72	Chlaenius	371.801
Lionepha	243.548	Lionepha	339.515	Lionepha	382.732	Lionepha	369.913
Omoglymmius	239.548	Omoglymmius	347.784	Omoglymmius	384.351	Omoglymmius	386.532
Pterostichus	234.219	Pterostichus	338.028	Pterostichus	383.419	Pterostichus	369.457
Trachypachus	240.534	Trachypachus	341.975	Trachypachus	379.898	Trachypachus	375.546
Total	1684.62	Total	2396.017	Total	2670.079	Total	2621.384

Taxon loci recovered in

Base genome	Omoglymmius		Pterostichus +1 taxon		Pterostichus +6 taxa		Trachypachus
Amphizoa	252.126	Amphizoa	716.109	Amphizoa	474.35	Amphizoa	343.923
Bembidion	246.201	Bembidion	687.505	Bembidion	450.986	Bembidion	334.553
Chlaenius	245.491	Chlaenius	688.296	Chlaenius	449.184	Chlaenius	338.172
Lionepha	244.87	Lionepha	698.058	Lionepha	453.472	Lionepha	333.889
Omoglymmius	252.094	Omoglymmius	746.155	Omoglymmius	500.936	Omoglymmius	343.596
Pterostichus	245.692	Pterostichus	703.528	Pterostichus	467.735	Pterostichus	336.919
Trachypachus	249.532	Trachypachus	700.501	Trachypachus	460.651	Trachypachus	337.36
Total	1736.008	Total	4940.155	Total	3257.31	Total	2368.415

Number of 'good loci' (UCE loci that appeared to be single copy across all taxa across all base genomes) recovered in different taxa from probe sets designed using different base genomes.

Supporting Data 9. Relative locus length of 'good loci'

Counts of the number of loci where that base genome led to the longest alignment (in up to 7 taxa), restricted to loci found in all 7 taxa (across a combination of base genomes)

Of the 2702 good loci found across all 7 taxa: Base genomes --->

Number of taxa base genome gave longest alignment in ↓	insilico-Amphizoa-base/ucelocus.txt	insilico-Bembidion-base/ucelocus.txt	insilico-Chlaenius-base/ucelocus.txt	insilico-Lionepha-base/ucelocus.txt	insilico-Omoglymmius-base/ucelocus.txt	insilico-Pterostichus+1-base/ucelocus.txt	insilico-Pterostichus+6-base/ucelocus.txt	insilico-Trachypachus-base/ucelocus.txt	
0	1860	1577	1564	1739	2055	1200	2250	1944	
1	161	185	233	226	183	326	80	258	By rows
2	177	211	181	171	124	217	39	160	
3	161	237	206	171	120	193	27	119	
4	142	179	169	140	86	192	21	102	
5	97	136	146	109	58	143	27	69	
6	62	108	115	89	43	129	26	32	
7	42	69	88	57	33	302	232	18	
	Amphizoa	Bembidion	Chlaenius	Lionepha	Omoglymmius	Pterostichus+1	Pterostichus+6	Trachypachus	
	insilico-Amphizoa	insilico-Bembidior	insilico-Chlaenius-b	insilico-Lionepha-	insilico-Omoglymı	insilico-Pterostic	insilico-Pterostich	insilico-Trachypachus-base/ucelocus	

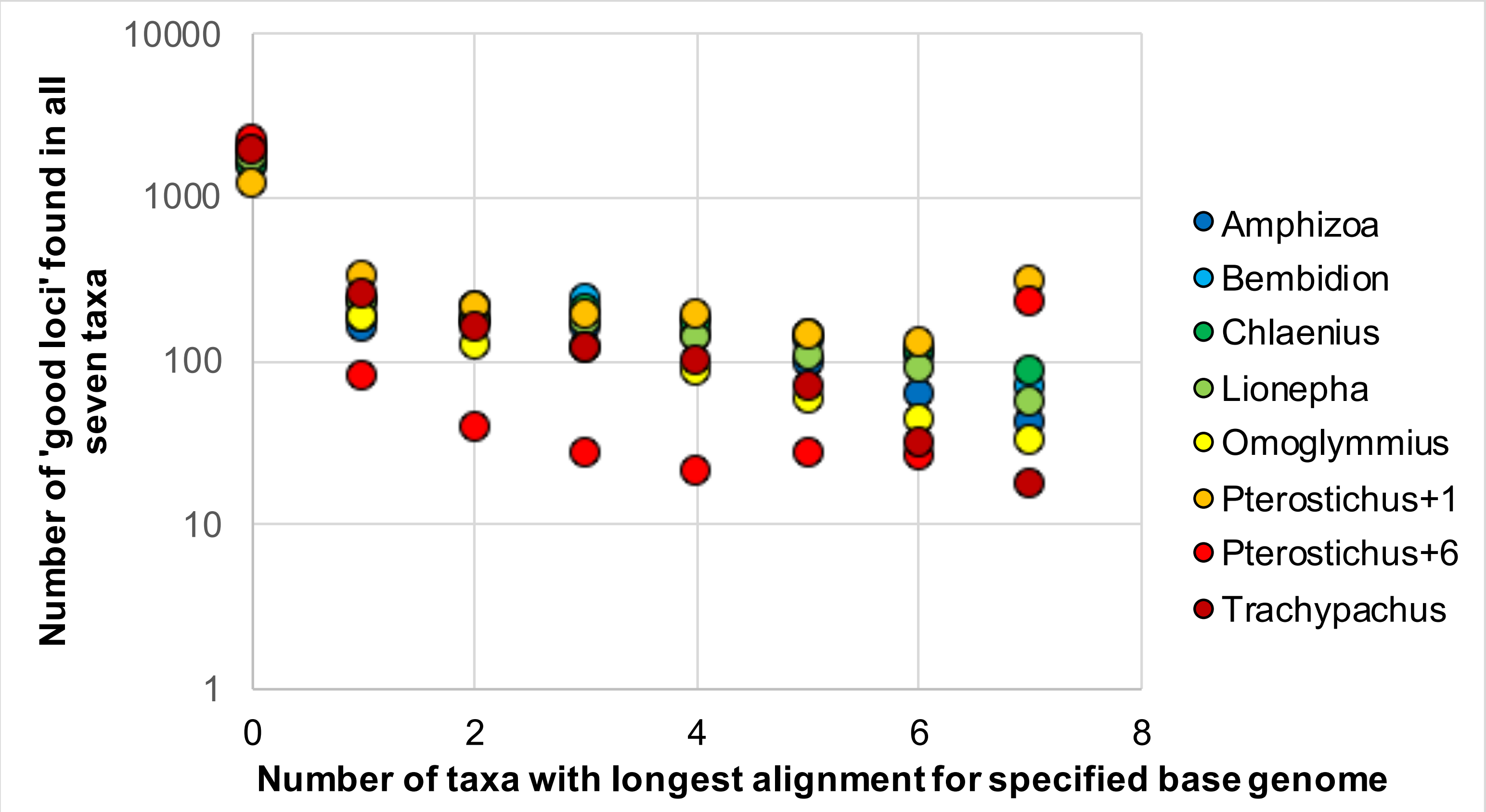

Supporting Data 10. Full genomic assembly metrics and BUSCO

Species	Voucher	#Contigs	Total bases	N50	L50	N90	L90
Bembidion haplogonum	DNA2544	1,376,511	665,124,290	591	211,683	231	972,875
Chlaenius sericeus	DNA4821	294,002	306,665,434	1,765	41,747	408	190,237
Lionepha "Waterfalls" sp.	DNA3782	107,315	185,440,171	5,477	9,102	518	47,974
Pterosticus melanarius	DNA3787	1,150,348	336,269,055	346	216,023	137	864,146
Omoyglymmius hammatus	DNA3783	1,292,279	397,439,551	370	234,264	143	963,963
Trachypachus gibbsii	DNA3786	425,659	279,738,584	1,758	34,686	213	233,955
Amphizoa insolens	DNA3784	894,291	270,779,264	364	160,074	141	668,749

Species	Voucher	%Adenine	%Cytosine	%Guanine	%Thymine	%N	GC%
Bembidion haplogonum	DNA2544	34.4	15.3	15.3	34.4	0.6	30.6
Chlaenius sericeus	DNA4821	35.1	14.5	14.5	35.1	0.8	29
Lionepha "Waterfalls" sp.	DNA3782	35.4	14.3	14.3	35.4	0.6	28.6
Pterosticus melanarius	DNA3787	34.1	34.1	15.9	15.9	0.1	48.8
Omoyglymmius hammatus	DNA3783	35.3	35.3	14.7	14.7	0	50
Trachypachus gibbsii	DNA3786	33.2	16.4	16.4	33.2	0.7	32.8
Amphizoa insolens	DNA3784	33.9	16.2	16.1	33.8	0.1	32.3

Species	Voucher	BUSCO Summary	Complete	Fragmented	Missing
Bembidion haplogonum	DNA2544	C:59.3%[S:57.5%,D:1.8%],F:27.2%,M:13.5%,n:2442	1,446	664	332
Chlaenius sericeus	DNA4821	C:78.7%[S:78.2%,D:0.5%],F:15.6%,M:5.7%,n:2442	1,923	380	139
Lionepha "Waterfalls" sp.	DNA3782	C:68.5%[S:68.3%,D:0.2%],F:20.1%,M:11.4%,n:2442	1,673	491	278
Pterosticus melanarius	DNA3787	C:30.7%[S:30.7%,D:0.0%],F:33.5%,M:35.8%,n:2442	750	817	875
Omoyglymmius hammatus	DNA3783	C:7.3%[S:7.3%,D:0.0%],F:28.3%,M:64.4%,n:2442	178	690	1,574
Trachypachus gibbsii	DNA3786	C:45.8%[S:45.2%,D:0.6%],F:31.5%,M:22.7%,n:2442	1,119	770	553
Amphizoa insolens	DNA3784	C:34.7%[S:34.5%,D:0.2%],F:31.2%,M:34.1%,n:2442	848	761	833

Supporting Data 11. Average genetic distance rankings

Distance rankings w/o tree patristic distances		18S AVERAGE	28S AVERAGE	ARGK AVERAGE	CAD2 AVERAGE	CAD4 AVERAGE	WG AVERAGE	COI AVERAGE	TOTAL AVERAGE	TOTAL AVERAGE excl 18S
Pterostichus	AF002779.1	1.333333	3.3333333	3	2	1	3	1	**2.10**	2.2222222
Trachypachus	AF002808.1	4.666667	3	4	5.3333333	7	4.3333333	2	**4.33**	4.2777778
Chlaenius	AF012473.1	1.666667	5	7	1	2	4.3333333	3	**3.43**	3.7222222
Bembidion	AF012490.1	3	1	1	4.3333333	5.333333333	1.3333333	5	**3.00**	3
Omoglymmius	AF012520.1	7	7	5	6	4.333333333	7	7	**6.19**	6.0555556
Amphizoa	AJ318678.1	5.666667	6	6	6.3333333	5.333333333	6	6	**5.90**	5.9444444
Lionepha'	KY246684.1	4.666667	2.6666667	2	3	3	2	4	**3.05**	2.7777778

rankings with tree patristic distances		AVERAGE	AVERAGE	AVERAGE	AVERAGE	AVERAGE	AVERAGE	AVERAGE	**AVERAGE**	**\GE excl 18S**
Pterostichus	AF002779.1	1.25	3.5	3	2	1	3	1	**2.11**	2.25
Trachypachus	AF002808.1	4.5	3	4	5.75	7	4.5	2	**4.39**	4.375
Chlaenius	AF012473.1	1.75	5	7	1	2	4.25	3	**3.43**	3.7083333
Bembidion	AF012490.1	3	1	1	4.25	5.25	1.25	5	**2.96**	2.9583333
Omoglymmius	AF012520.1	7	7	5	5.75	4.25	7	7	**6.14**	6
Amphizoa	AJ318678.1	5.75	6	6	6.25	5.5	6	6	**5.93**	5.9583333
Lionepha sp.	KY246684.1	4.75	2.5	2	3	3	2	4	**3.04**	2.75

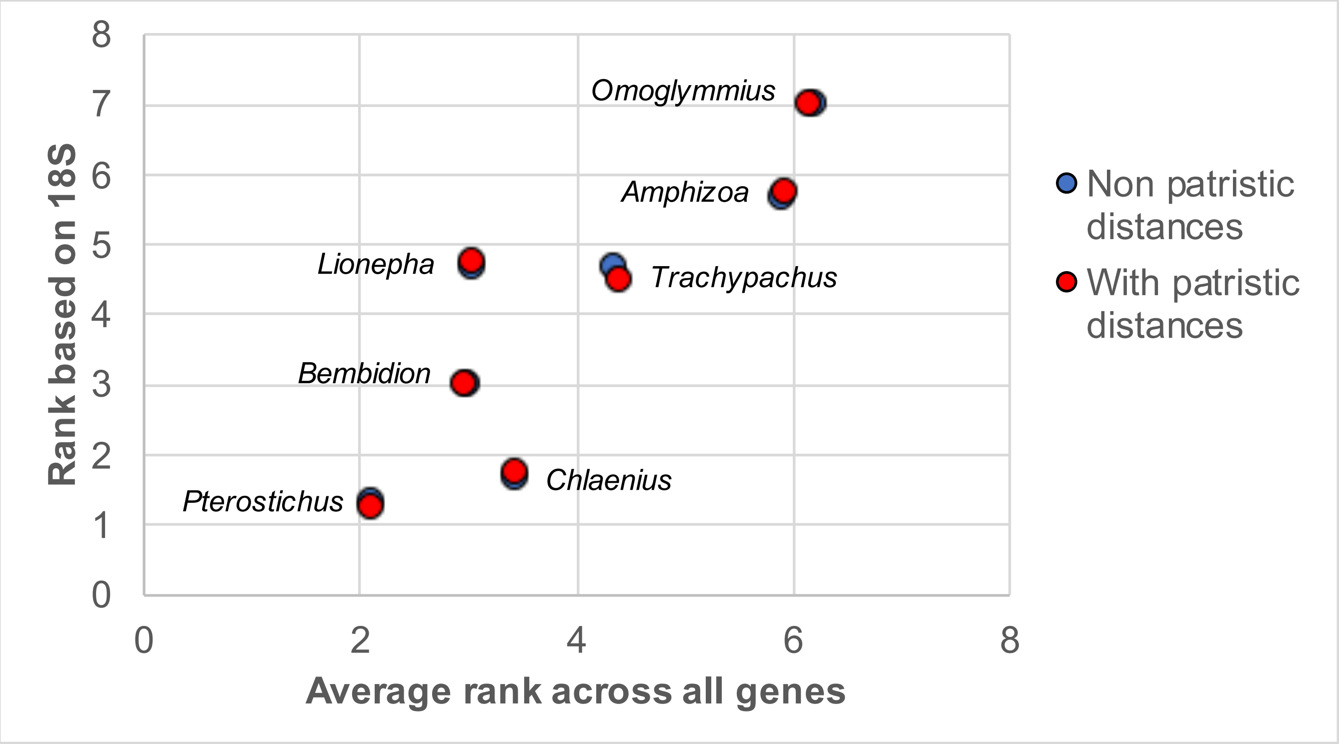

Supporting Data 11. Average genetic distance rankings

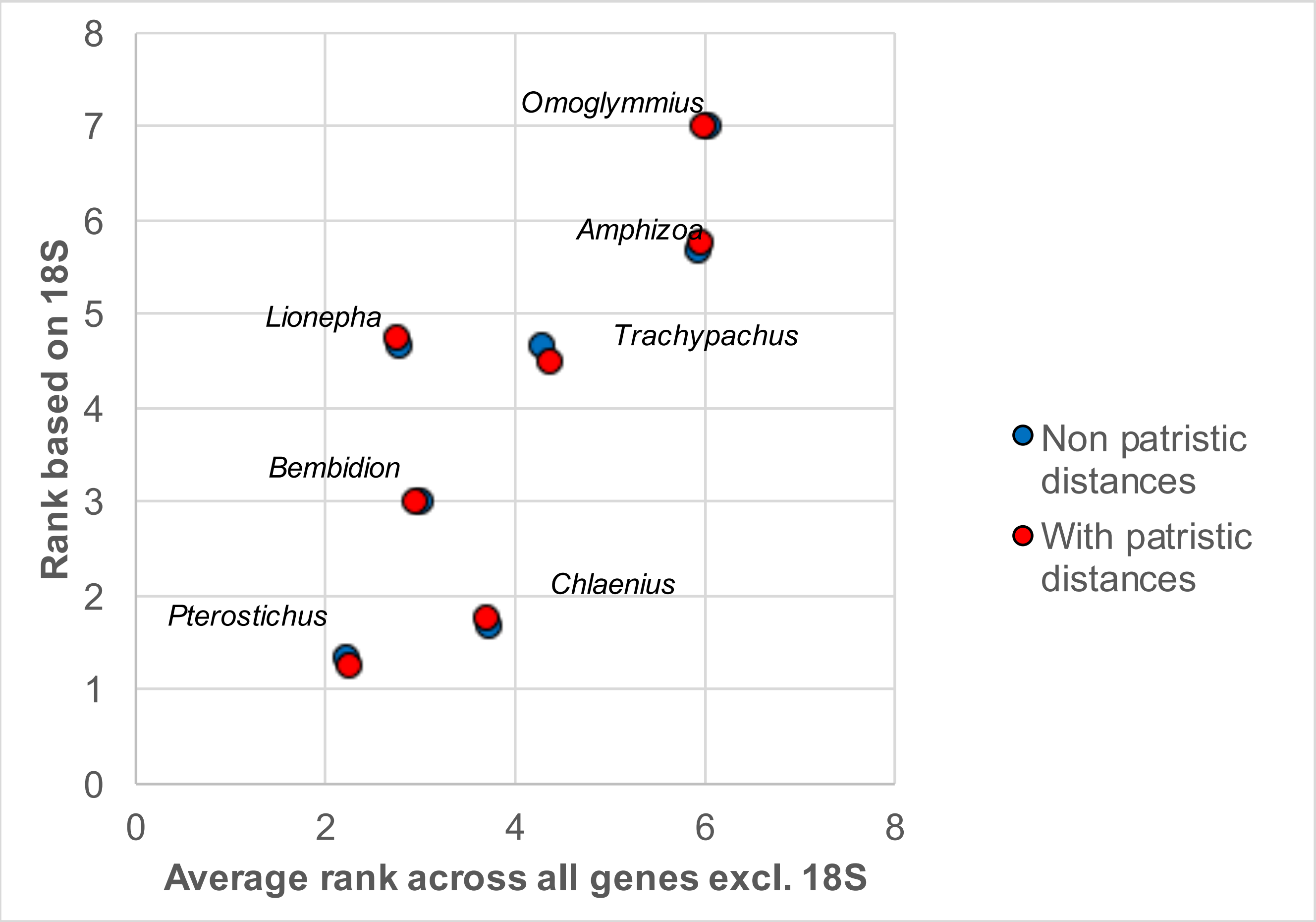

Supporting Data 12. Raw genetic distance per locus based on pairwise comparison of all seven species for six gene fragments commonly used in phylogenetic studies

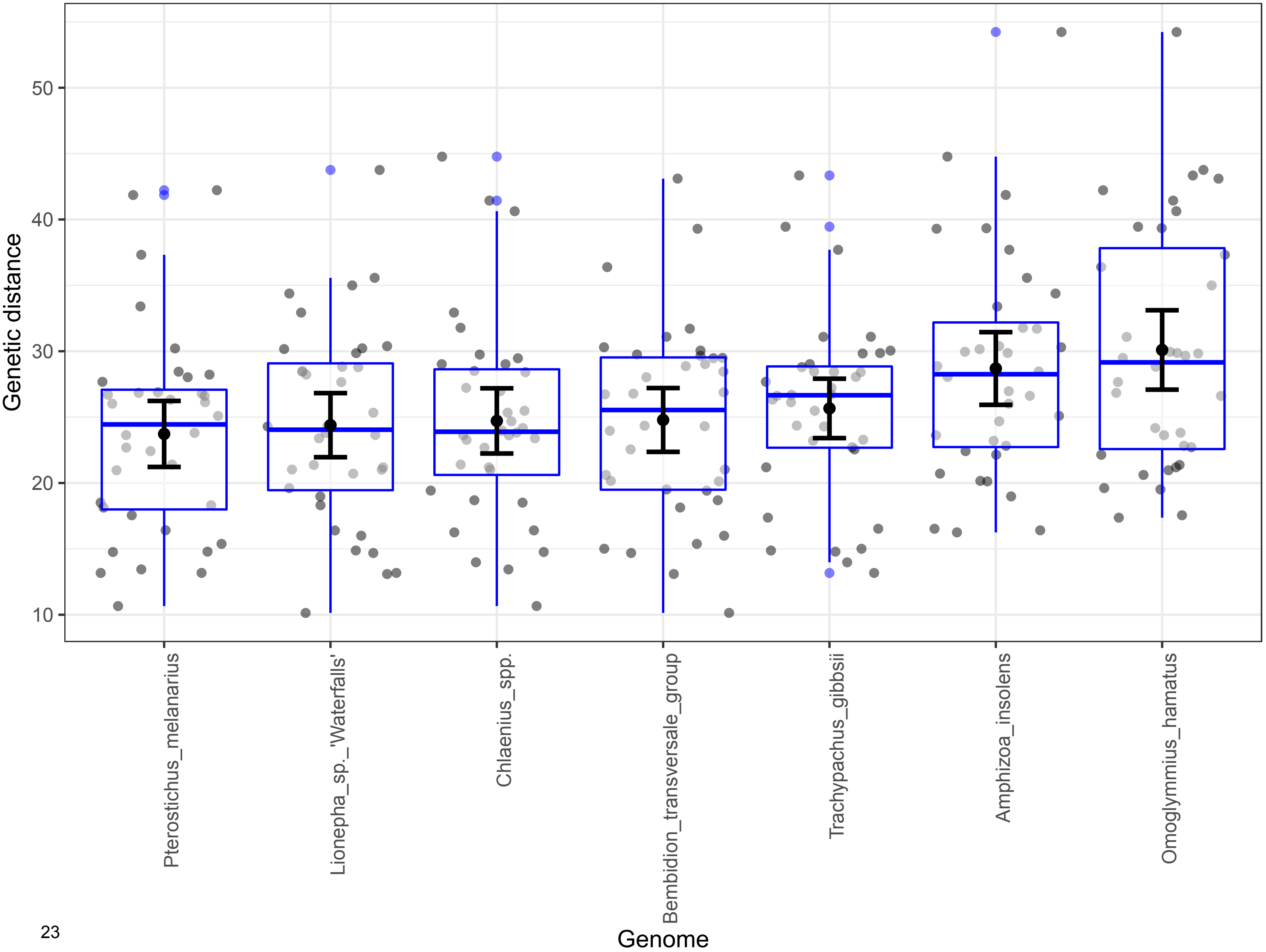

Supporting Data 13. Standardized genetic distance based on maximum distance per locus between any pair of the seven species for six gene fragments commonly used in phylogenetic studies

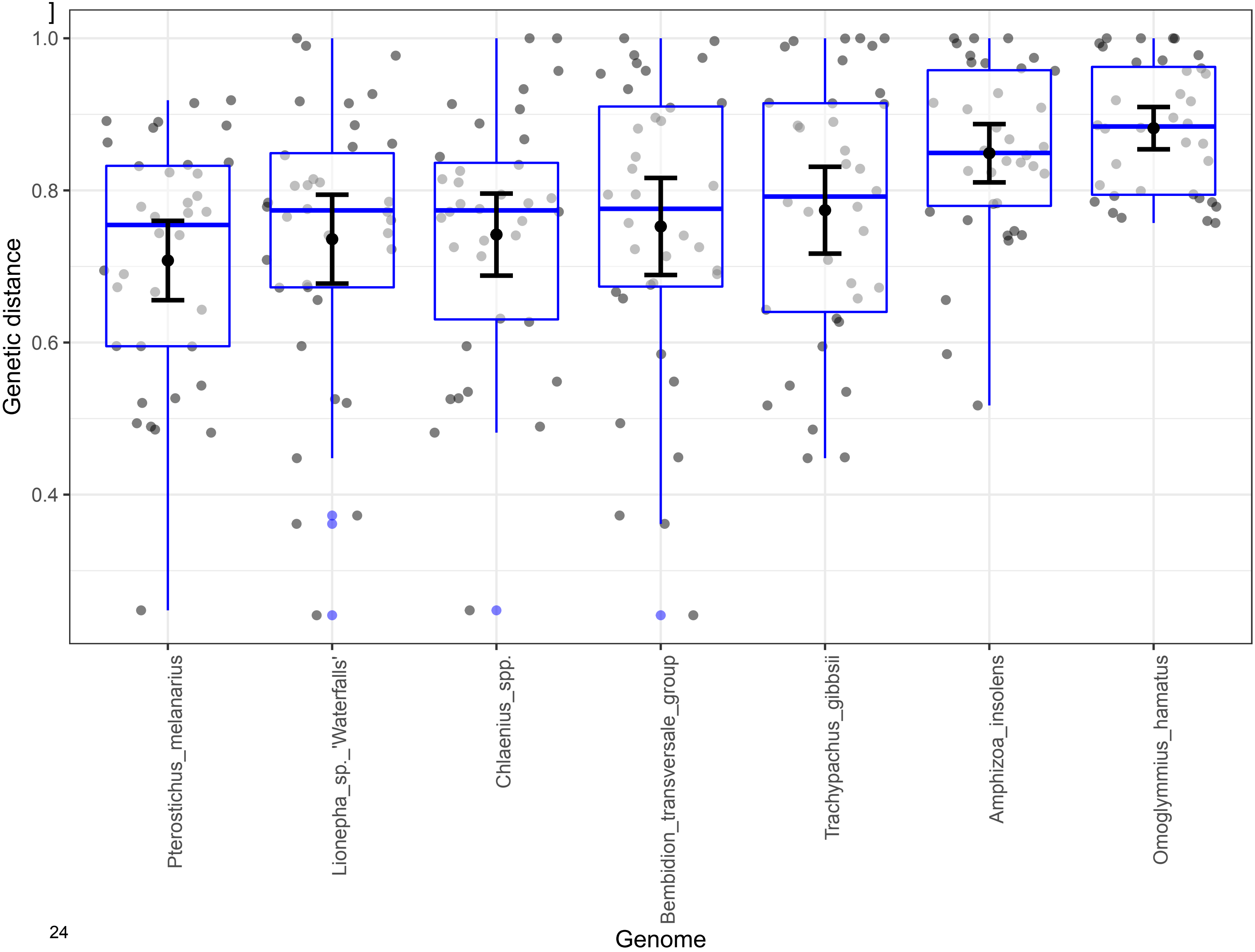

Supporting Data 14. Raw genetic distance per locus based on pairwise comparison of nuclear protein-coding loci extracted from genomic assemblies that were found across two or more taxa

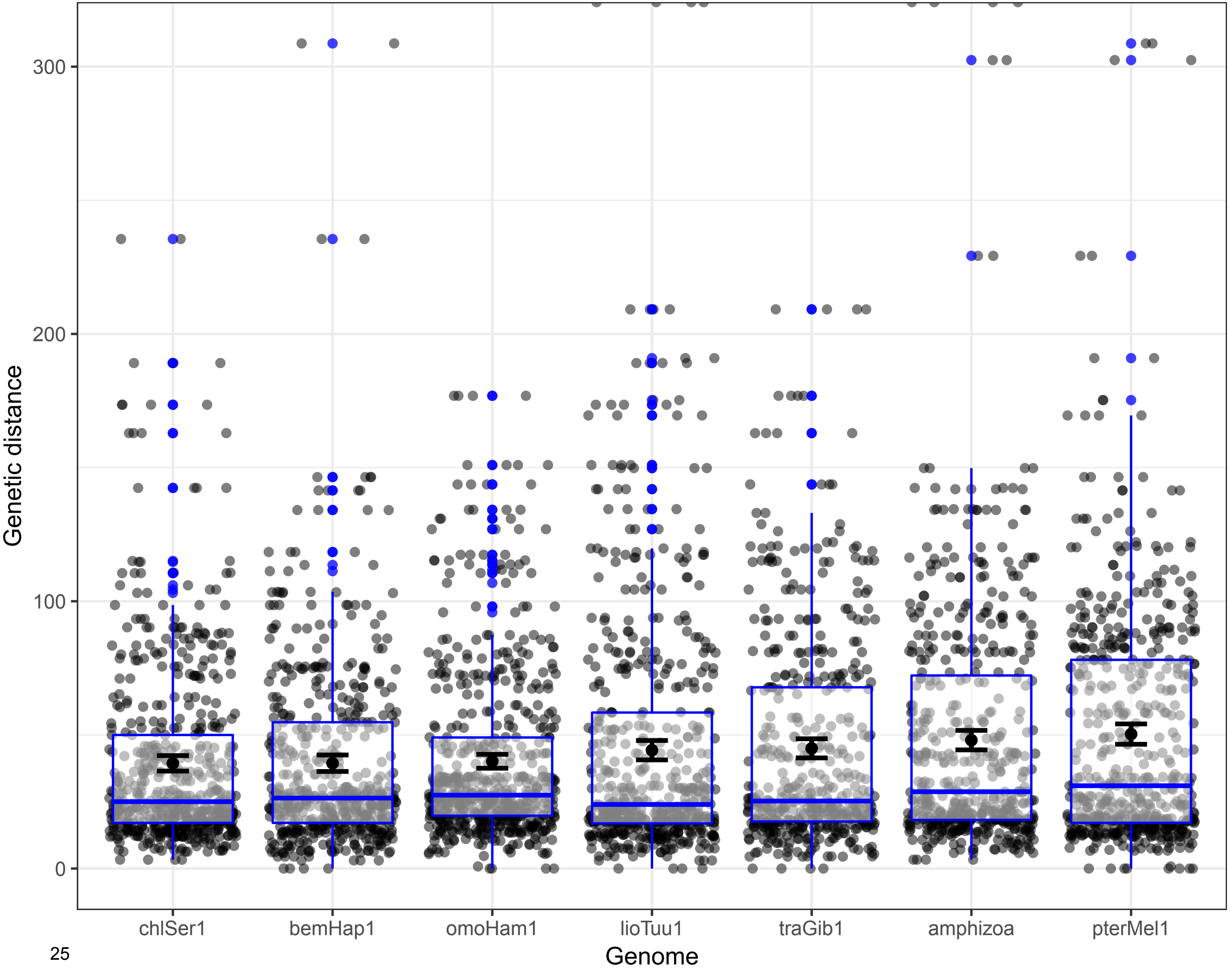

Supporting Data 15. Raw genetic distance per locus based on pairwise comparison of nuclear protein-coding loci extracted from genomic assemblies that were found across three or more taxa

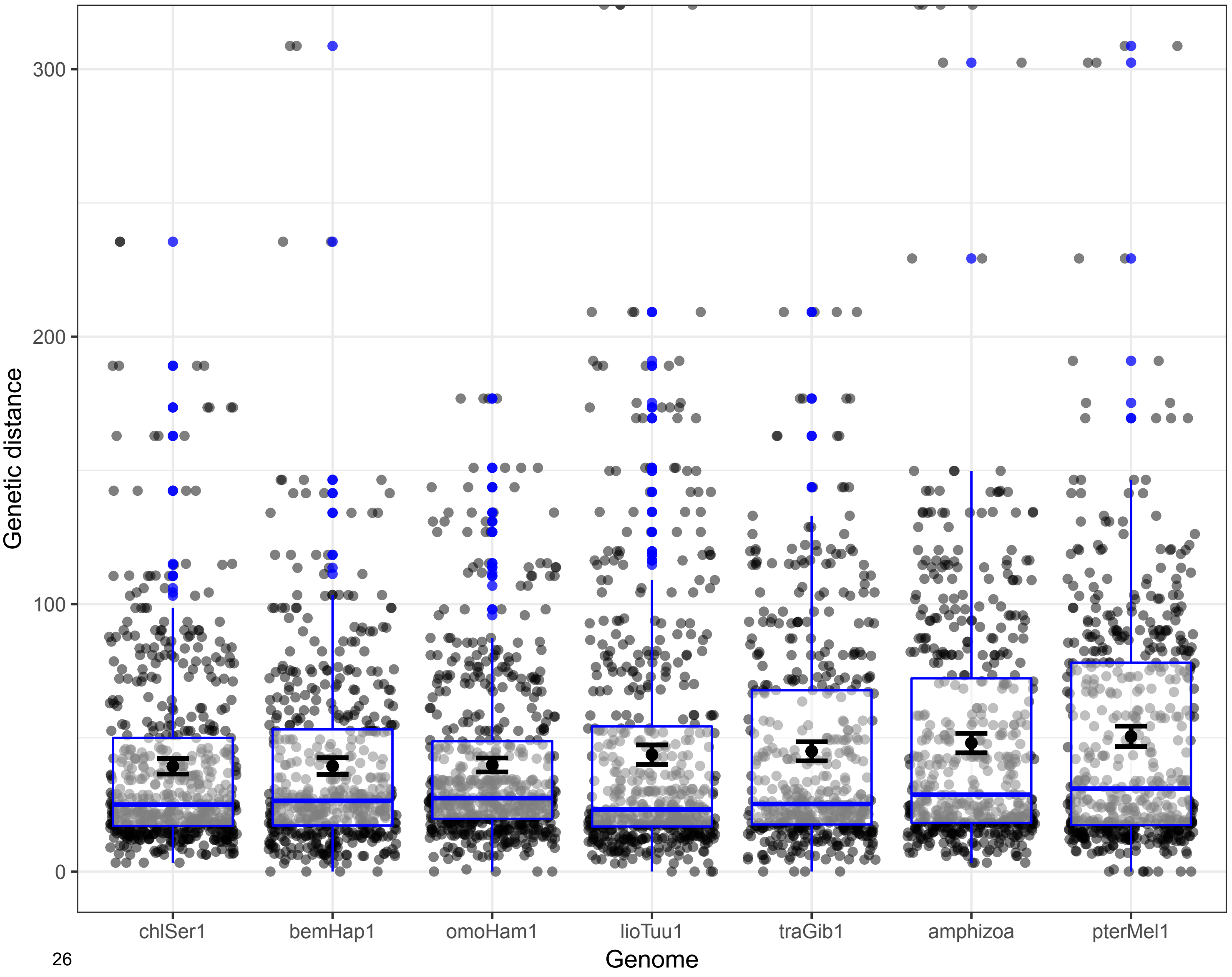

Supporting Data 16. Standardized genetic distance based on maximum pairwise distance per locus for nuclear protein coding loci extracted from genomic assemblies that were found across three or more taxa

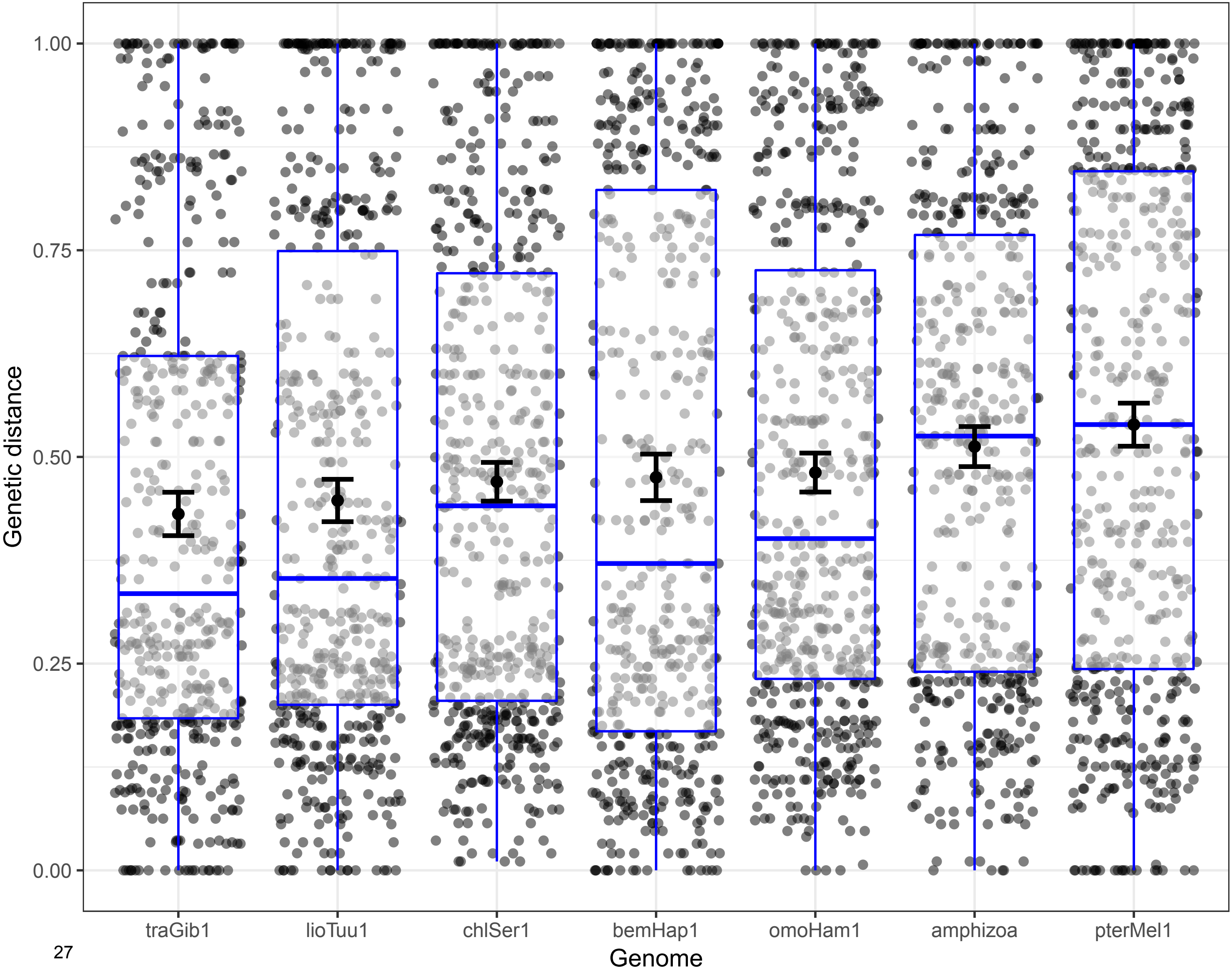

Supporting Data 17. Raw genetic distance per locus based on pairwise comparison of nuclear protein-coding loci extracted from genomic assemblies that were found across all seven taxa

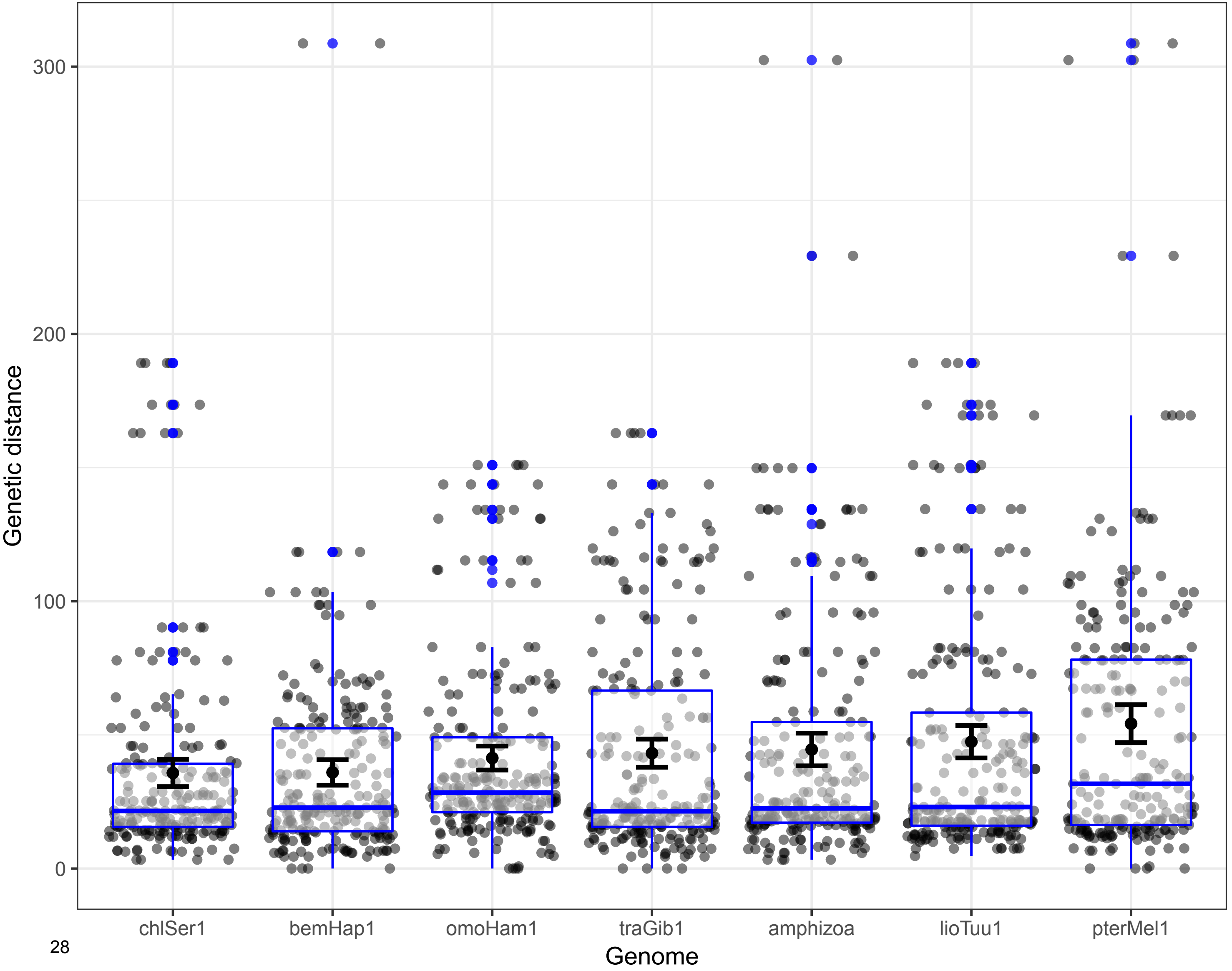

Supporting Data 18. Standardized genetic distance based on maximum pairwise distance per locus for nuclear protein coding loci extracted from genomic assemblies that were found across all seven taxa

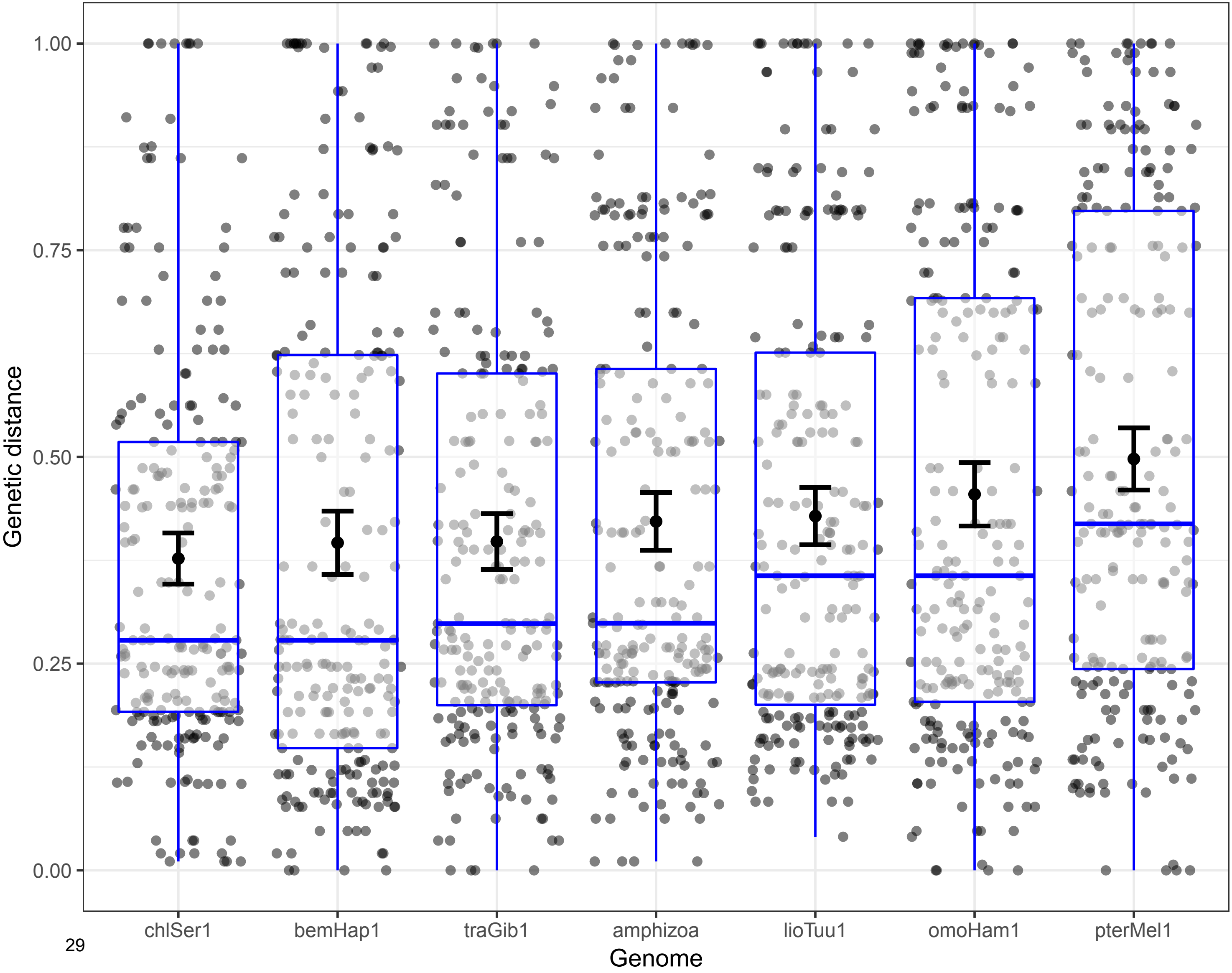

Supporting Data 19. Graphs showing the number of nuclear protein coding genes extracted directly from genomic assemblies, mean raw genetic distance and mean standardized genetic distances estimated from these loci, the locus length, as well as minimum and maximum coverage of the extracted genes.

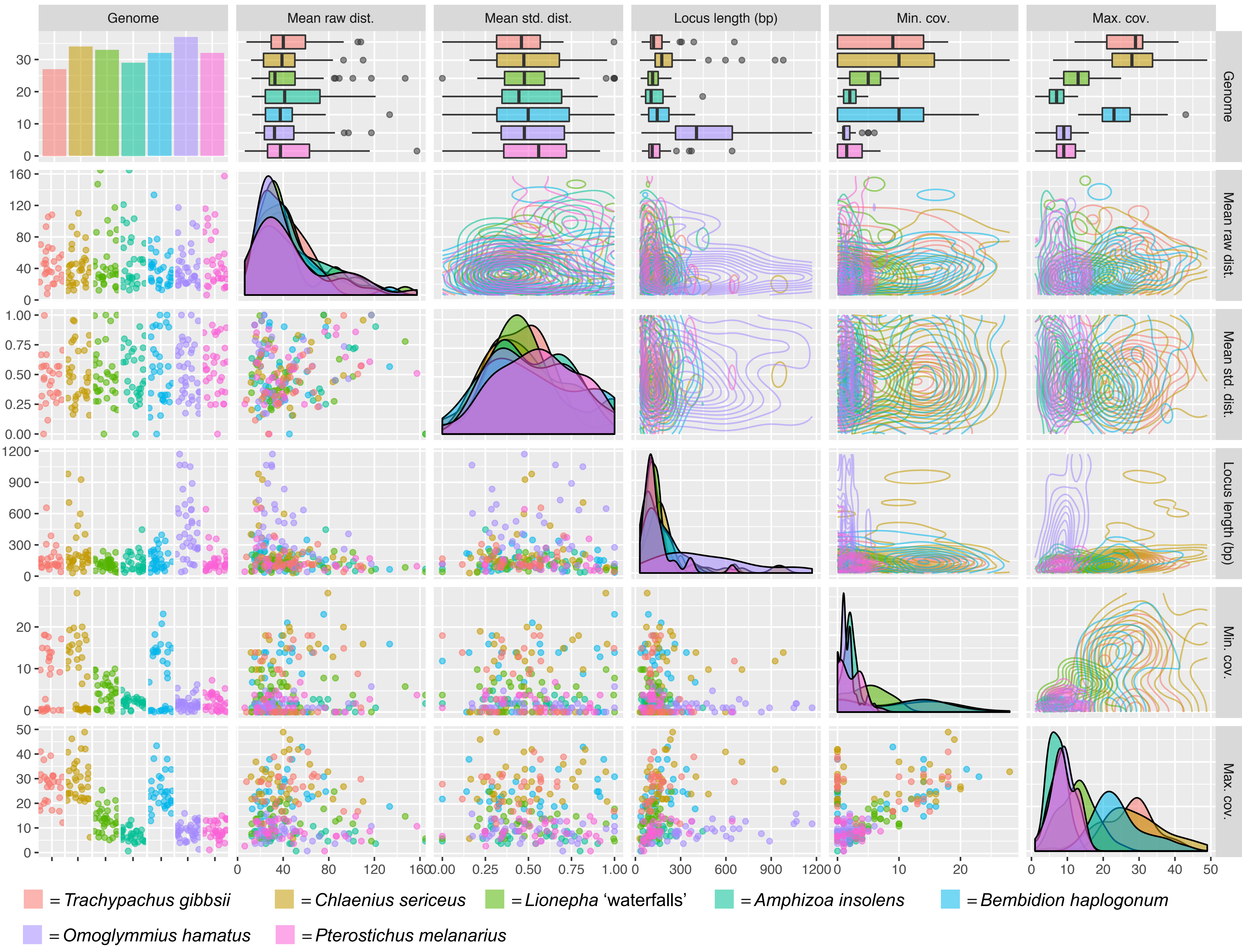

Supporting Data 20. Comparison of maximum likelihood phylogenies of loci used for calculating genetic distances

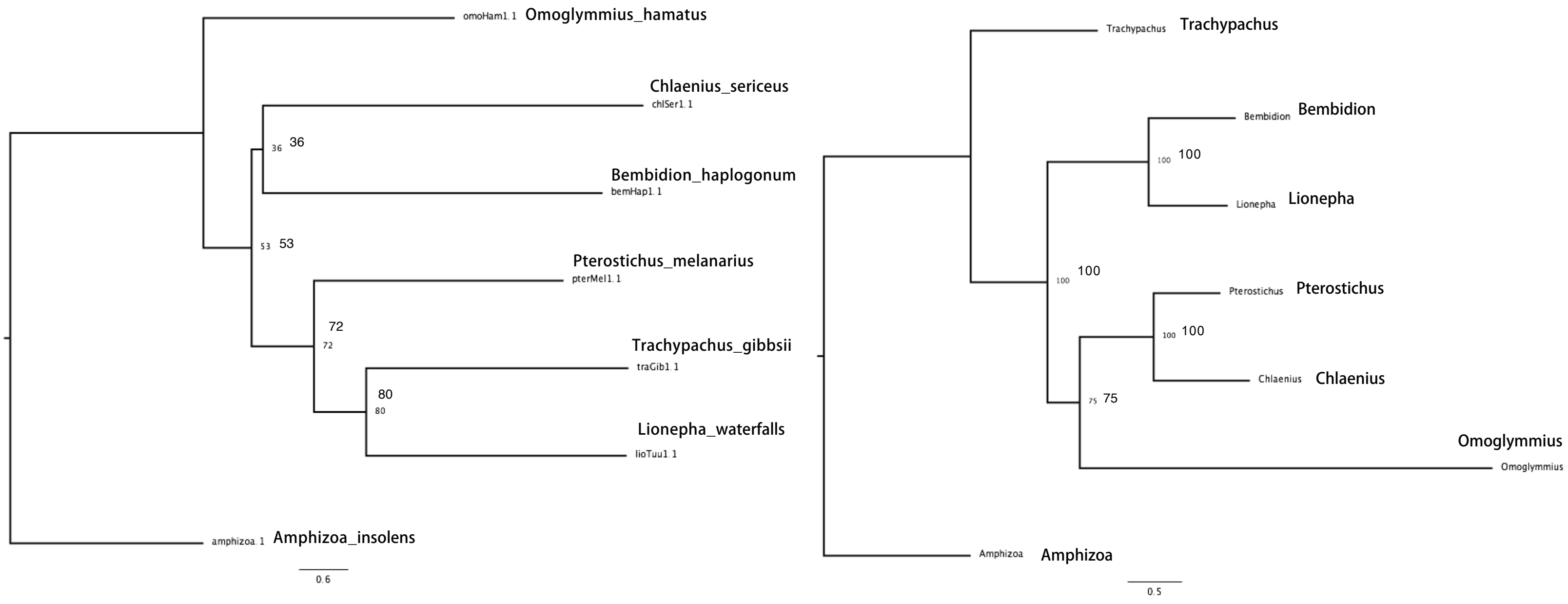

A **B**

Results of maximum likelihood analysis using IQTree under the settings -s [input_maxtrix.phy] -spp [input_gene_&_codon_partitions.nex] -m TESTMERGE -bb 1000 -nt 6 on (A 50 nuclear protein-coding markers extracted directly from genomic assemblies; and (B six gene fragments commonly used in phylogenetic studies.

The six Sanger-sequenced genes commonly used in phylogenetic studies on Coleoptera (28S, COI, CAD2, CAD4, wg, ArgK) (B, above) recovered previously well-established relationships (Maddison *et al.* 2009; Maddison 2012; McKenna *et al.* 2015) such as *Bembidion* and *Lionepha* as sister (in the same tribe, formerly same genus), *Chlaenius* and *Pterostichus* as sister (in the same subfamily), and the family Carabidae as a clade with the inclusion of *Omoglymmius* (but with low support values) when analyized in a maximum likelihood framework. The UCE data supported (Fig. S1) these same relationships with the exception of *Omoglymmius* being placed as sister to Carabidae with strong support. However, this alternative relationship is consistent with morphology as *Omoglymmius* is often regarded as a different family, Rhysodidae. In contrast, the 50 different nuclear protein-coding loci extracted directly from the genomic assemblies of our study taxa in order to provide additional measurements of genetic distance failed to recover any of these well-supported relationships (A, above). Additionally, *Trachypachus gibbsii* representing the family Trachypachidae was nested within the Carabidae clade, rendering it paraphyletic. There appears to be an evident correlation between the genetic distance measures (Supporting Data 14-18) of these loci and sequencing coverage of the genomes they were extracted from (Table 1, Supporting Data 19), suggesting the influence of sequencing error due to low depth of coverage and “missingness” of data. This would explain the strange topology recovered by the maximum likelihood analysis of these data compared to that recovered by both fewer (B, above) and more genetic loci (Fig. S1). As these loci appear suspect and likely affected by sequencing error, they are provided only in Supplemental File 1 and are not uploaded to GenBank.
